# Action observation reveals a network with divergent temporal and parietal cortex engagement in dogs compared with humans

**DOI:** 10.1162/imag_a_00385

**Published:** 2024-12-11

**Authors:** Magdalena Boch, Sabrina Karl, Isabella C. Wagner, Lukas L. Lengersdorff, Ludwig Huber, Claus Lamm

**Affiliations:** Social, Cognitive and Affective Neuroscience Unit, Department of Cognition, Emotion, and Methods in Psychology, Faculty of Psychology, University of Vienna, Vienna, Austria; Department of Cognitive Biology, Faculty of Life Sciences, University of Vienna, Vienna, Austria; Comparative Cognition, Messerli Research Institute, University of Veterinary Medicine Vienna, Medical University of Vienna and University of Vienna, Vienna, Austria

**Keywords:** comparative neuroscience, action observation network, social cognition, dogs, humans

## Abstract

Action observation is a fundamental pillar of social cognition. Neuroimaging research has revealed a human and non-human primate action observation network (AON) encompassing frontotemporoparietal areas with links to the species’ imitation tendencies and relative lobe expansion. Dogs (*Canis familiaris*) have good action perception and imitation skills and a less expanded parietal than temporal cortex, but their AON remains unexplored. We conducted a functional MRI study with 28 dogs and 40 humans and found functionally analogous involvement of somatosensory and temporal brain areas of both species’ AONs and responses to transitive and intransitive action observation in line with their imitative skills. Employing a functional localizer, we also identified functionally analogous agent-responsive areas within both species’ AONs. However, activation and task-based functional connectivity measures suggested significantly less parietal cortex involvement in dogs than in humans. These findings advance our understanding of the neural bases of action understanding and the convergent evolution of social cognition, with analogies and differences resulting from similar social environments and divergent brain expansion, respectively.

## 1 Introduction

The actions of other individuals contain a wealth of social information. For example, we may use them to infer others’ intentions (Blakemore & Decety, 2001) or learn a new skill by imitating their actions (Heyes, 2021). Perceiving others’ actions also plays an integral part in social interactions, such as cooperation (Endedijk et al., 2017) and, therefore, may foster long-term social relationships.

Neuroimaging research in humans has consistently revealed that the observation of transitive (i.e., goal-directed) and intransitive (i.e., goal-absent) actions is underpinned by the action observation network (AON; see e.g.,Caspers et al., 2010;Hardwick et al., 2018for meta-analysis or review). This distributed network comprises the inferior occipitotemporal ventral visual pathway housing face- and body-sensitive areas and a lateral occipitotemporal pathway associated with the perception of dynamic aspects of social cues and action features (Pitcher & Ungerleider, 2021;Ungerleider & Haxby, 1994;Wurm & Caramazza, 2022for reviews). The parietal dorsal visual pathway, including especially the inferior parietal lobule, is also part of the AON and associated with “vision-to-action” brain functions that guide reach-to-grasp actions (seeGlover et al., 2012orUngerleider & Haxby, 1994for review). Finally, the AON involves sensory–motor areas, such as the premotor cortex, which, as the parietal regions, are also active during the observation and execution of the same action.

A network similar to the human AON is also present in nonhuman primates. This network, however, shows species differences that provide important insights into the evolution of the human AON. In rhesus macaques (*Macaca mulatta*) and chimpanzees (*Pan troglodytes*), action observation elicits the strongest activation in frontoparietal regions (Hecht, Murphy, et al., 2013;Sliwa & Freiwald, 2017), but common marmosets (*Callithrix jacchus*) have greater frontotemporal activation (Zanini et al., 2023). In Old World monkeys, apes, and humans, the frontal and parietal lobes expanded disproportionally compared with the other lobes. In contrast, other primate (including marmosets) and mammalian species have more expanded occipital and temporal lobes (Garin et al., 2022). Thus, relative lobe expansions might be linked to differences in activation patterns and, consequently, brain and cognitive functions across these primate species.

The distributed activation pattern in the human AON has been linked to more pronounced connections (i.e., white matter pathways) between the parietal and especially the inferior temporal cortex, relative to chimpanzees and macaques, where these connections are the least pronounced of the three species (Hecht, Gutman, et al., 2013). In humans and chimpanzees, but not in macaques, the AON is similarly active during both transitive and intransitive action observation (Caspers et al., 2010;Fiave & Nelissen, 2021;Hecht, Murphy, et al., 2013;Nelissen et al., 2011). The differences in structural connectivity and activation for transitive and intransitive actions have been associated with the species’ ability to learn tool-using behaviours through social learning and, in particular, their tendency to imitate actions, which has been associated with a stronger focus on encoding the action rather than the outcome or goal (Hecht, Gutman, et al., 2013). Humans and (less successfully) chimpanzees can acquire tool use through social learning and imitating actions, but macaques cannot as they are emulators focusing on the action outcome (Hecht, Gutman, et al., 2013;Mitchell & Anderson, 1993;Pope et al., 2018;Whiten et al., 2009). Recent findings in marmosets (Zanini et al., 2023), which are not known as tool users either but display true imitation of behaviours (Voelkl & Huber, 2000,^2007^), make the suggested relationship between comparable activation during transitive and intransitive action observation and the tendency to imitate actions of others less clear. Here, both types of actions elicited activation in the entire AON but with stronger activation for transitive than intransitive actions in premotor, parietal, and temporal regions. However, activation in the temporal cortex was much more pronounced than in the parietal cortex, and the findings did not indicate stronger engagement of the inferior parietal lobule (IPL), a brain area typically associated with goal representation (Fiave & Nelissen, 2021;Nelissen et al., 2011).

Another approach to studying the human AON’s evolutionary history is to search for convergent evolution in a different animal lineage. So far, research in non-primate species has focused on investigations of mirror neurons in the motor and anterior cingulate cortex of rats (Carrillo et al., 2019;Viaro et al., 2021) and in the forebrain of various songbirds (Fujimoto et al., 2011;Hamaguchi et al., 2014;Prather et al., 2008). However, due to the nature of these studies’ electrophysiological recordings, they could not explore whole-brain networks that underpin action observation and thus remain agnostic to putative further functional analogies with humans or non-human primates. Dogs (*Canis familiaris*) represent a highly relevant additional species for comparative research on action observation for several reasons. First, they do not engage in tool-using behaviours but demonstrate good action perception and imitation skills. For example, dogs can distinguish between actions that reflect different intentions (Völter, Lonardo, et al., 2023; and seeHuber & Lonardo, 2023for review), they can imitate actions of dogs and humans (Fugazza & Miklósi, 2014;Kubinyi et al., 2003;Range et al., 2007,^2011^;Topál et al., 2006), and even over-imitate actions demonstrated by their primary human caregivers (Huber et al., 2018,^2020^,^2022^). Second, beyond the preserved subcortical areas such as the amygdala or hippocampus, dogs have a temporal cortex that evolved in carnivorans independently of primates (Bryant & Preuss, 2018;Kaas, 2011;Lyras, 2009). However, like marmosets, dogs do not have a significantly expanded parietal cortex (Garin et al., 2022). Third, they can be trained to participate awake and unrestrained in functional MRI studies (Karl et al., 2020), and dogs attend and react to stimuli presented on a computer screen (Völter & Huber, 2021;Völter, Tomašić, et al., 2023;Völter et al., 2020). This allows us to conduct comparative studies on dogs and humans using largely identical scanning conditions. Hence, dogs constitute an excellent model for studying the evolutionary history of the human AON and the relationship with social learning, tool-use behaviours, and differential lobe expansion.

In the present comparative functional MRI study, we thus performed the first investigation of the dog action observation network (AON). We aimed to identify functional analogies and divergencies between the dog and human AONs by applying univariate activation and task-based functional connectivity analyses (McLaren et al., 2012;O’Reilly et al., 2012). We used experimental manipulations that allowed us to assess AON engagement to intransitive versus transitive actions and actions performed by conspecifics versus heterospecifics and controlled for low-level visual aspects.

To identify potential functional analogues between the two species, we first assessed activation in brain areas involved in sensory–motor processes in both species (e.g., dog precruciate and postcruciate gyrus and human inferior frontal gyrus; seeFig. 3Afor details, andBoch et al., 2024, for a review of dog sensory areas). Second, we hypothesized that the dog action observation network, as in humans, includes occipitotemporal brain areas associated with face and body perception (i.e., agent areas), as well as areas involved in the processing of dynamic aspects of social cues and action features (Pitcher & Ungerleider, 2021;Ungerleider & Haxby, 1994;Wurm & Caramazza, 2022). First evidence suggests that the dog agent areas are housed in the occipitotemporal ectomarginal as well as the mid and caudal suprasylvian gyrus, but results have been mixed (Boch et al., 2023b;Bunford et al., 2020;Cuaya et al., 2016;Dilks et al., 2015;Gillette et al., 2022;Hernández-Pérez et al., 2018;Szabó et al., 2020;Thompkins et al., 2018). Brain areas associated with the processing of dynamic aspects have yet to be investigated. Thus, to test our hypothesis, we used a functional localizer task to identify the agent areas within the dog AON and disentangle them from areas sensitive to the action features. Third, in light of the species’ imitation and action-matching skills (Fugazza et al., 2023;Hoehl et al., 2019;Huber et al., 2020;Range et al., 2007), we predicted comparable activation of the dog and human action observation networks while observing transitive and intransitive actions. Fourth, regarding their close bond, we also explored how the species’ action observation network responded to actions performed by the other species versus conspecifics. However, we also expected functional divergencies between the dog and human AON based on prior comparative research with non-human primates (Hecht, Murphy, et al., 2013;Sliwa & Freiwald, 2017;Zanini et al., 2023). Considering differential relative lobe expansion in dogs and humans (Garin et al., 2022), we predicted the strongest engagement of the dog temporal cortex during action observation and less parietal cortex involvement than in humans. We quantitatively tested this hypothesis by first comparing the extent of active voxels in both species’ parietal and temporal cortex during action observation. Then, we also investigated task-based functional connectivity between the primary visual cortex (V1) and each lobe to investigate a potentially greater information exchange with the temporal than parietal cortex in dogs.

## 2 Methods

### 2.1 Participants

#### 2.1.1 Action observation task

Twenty-eight pet dogs (*Canis familiaris*; 12 females, age range: 2–12 years, mean age: 6.1 years), extensively trained to undergo MRI scanning (Karl et al., 2020), participated in the study. More than half of the sample were pure-bred breeds (9 Border Collies, 5 Australian Shepherds, 1 Labrador Retriever, 1 Nova Scotia Duck Tolling Retriever, 1 Border Collie Australian Shepherd mix, 1 Labrador retriever mix, 1 Small Munsterlander, 1 Leisha dog, 8 mixed breeds). The dogs’ weight ranged from 16 to 29 kg (mean = 21.58 kg,*SD*= 4.77). The data collection period lasted from May 2020 to March 2023, and we set the minimum sample size to*N*= 12 dogs, which was the median sample size of awake dog fMRI studies at the time of planning this study and still roughly was in 2023 (i.e., by the time of submitting this study). All dogs underwent a veterinary medical check before data collection to assess their general health and eyesight, and the human caregiver gave informed written consent to their participation.

We collected comparative data from*N*= 40 human participants (22 females, age range: 19–28 years, mean age: 23 years) who also participated in an fMRI study investigating face and body perception in dogs and humans (Boch et al., 2023b) in the same session (data collection period: September to November 2020). We determined the sample size based on previous studies in our laboratory and other comparative neuroimaging studies (Bunford et al., 2020) with similar task designs. Human participants were right handed with normal or corrected-to-normal vision; they had no history of neurological or psychiatric diseases, did not report fear of dogs and gave informed written consent.

#### 2.1.2 Agent localizer

Findings so far about the location of face- and body-sensitive areas in the dog brain were mixed, and a lack of shared template space makes it even more challenging to build on prior work. We, therefore, additionally used a functional localizer task for the dogs, which allowed us to detect functional analogues of the human ventral temporal pathway (i.e., housing face- and body-sensitive areas;Pitcher & Ungerleider, 2021;Ungerleider & Haxby, 1994) within the canine action observation network within our sample. The agent localizer sample comprises*N*= 28 pet dogs (15 females, age range: 2–10, mean age: 5.5 years). Twenty-four of these dogs also participated in the main task on action observation, and data from*n*= 15 dogs were published as part of the original publication of our group investigating agent perception in dogs and humans (Boch et al., 2023b).

Detailed sample descriptives are openly available at the study’s OSF data repository. Dog data collection was approved by the institutional ethics and animal welfare commission in accordance with Good Scientific Practice (GSP) guidelines and national legislation at the University of Veterinary Medicine Vienna (ETK-06/06/2017), based on a pilot study conducted at the University of Vienna. The comparative human data collection was approved by the ethics committee of the University of Vienna (reference number: 00565) and performed in line with the latest revision of the Declaration of Helsinki (2013).

### 2.2 Experimental design

#### 2.2.1 Action observation task

In two 5-min task runs, dog and human participants saw videos of a dog or human agent grasping a ball (transitive action) or a video of which the ball was edited out (intransitive action, i.e., identical movement kinematics but no visible action goal). They also saw videos with the agent edited out (object motion condition) to control for object motion with the same trajectory and velocity as in the transitive action video and a phase-scrambled version of the transitive action video serving as a low-level visual and motion characteristics control (seeSection 2.2.4for details). Thus, participants saw six different conditions: dog transitive actions, human transitive actions, dog intransitive actions, human intransitive actions, object motion, and phase-scrambled control (seeFig. 1C). Half of the control condition blocks showed object and scrambled motion based on the human and the other half based on the dog transitive action videos. We presented the videos in a block design (duration: ~12 s; 4 different videos per block), interspersed with a visual baseline (3–7 s jitter) depicting a white cross on grey background using Psychopy (Peirce, 2007). We chose a block design because it is also commonly used for primate action observation localizers (see e.g.,Nelissen et al., 2005,^2011^;Sliwa & Freiwald, 2017) and due to the greater statistical power and robustness compared with event-related designs. Participants saw three blocks per condition in randomized order in each run, but the same condition was only presented once in a row (i.e., 18 blocks per task run). Video composition for each block and order within blocks were randomized across participants. Dogs and human participants were trained or instructed to attend to the MR compatible screen (32 inch) placed at the end of the scanner bore (passive viewing paradigm). Dogs viewed the task in sphinx position (seeFig. 1A); to ensure they could see the videos without looking up or moving their head and to minimize the need to perform frequent eye movements, we presented the videos and implicit baseline at their eye level (i.e., 100 pixels below the centre of the digital screen), resized to 750 × 750 pixels. Humans viewed the task in a supine position via a mirror placed on the head coil, with videos presented at the centre of the MR screen at their eye level. The distance between the eyes and the MR screen was approximately 85 cm for the dogs and 102 cm for humans (92 cm from the MR screen to the mirror + 10 cm from the mirror to the eyes). The dogs’ eyes were slightly closer because they could look directly at the screen through the k9 head coil (seeFig. 1A).

**Fig. 1.**
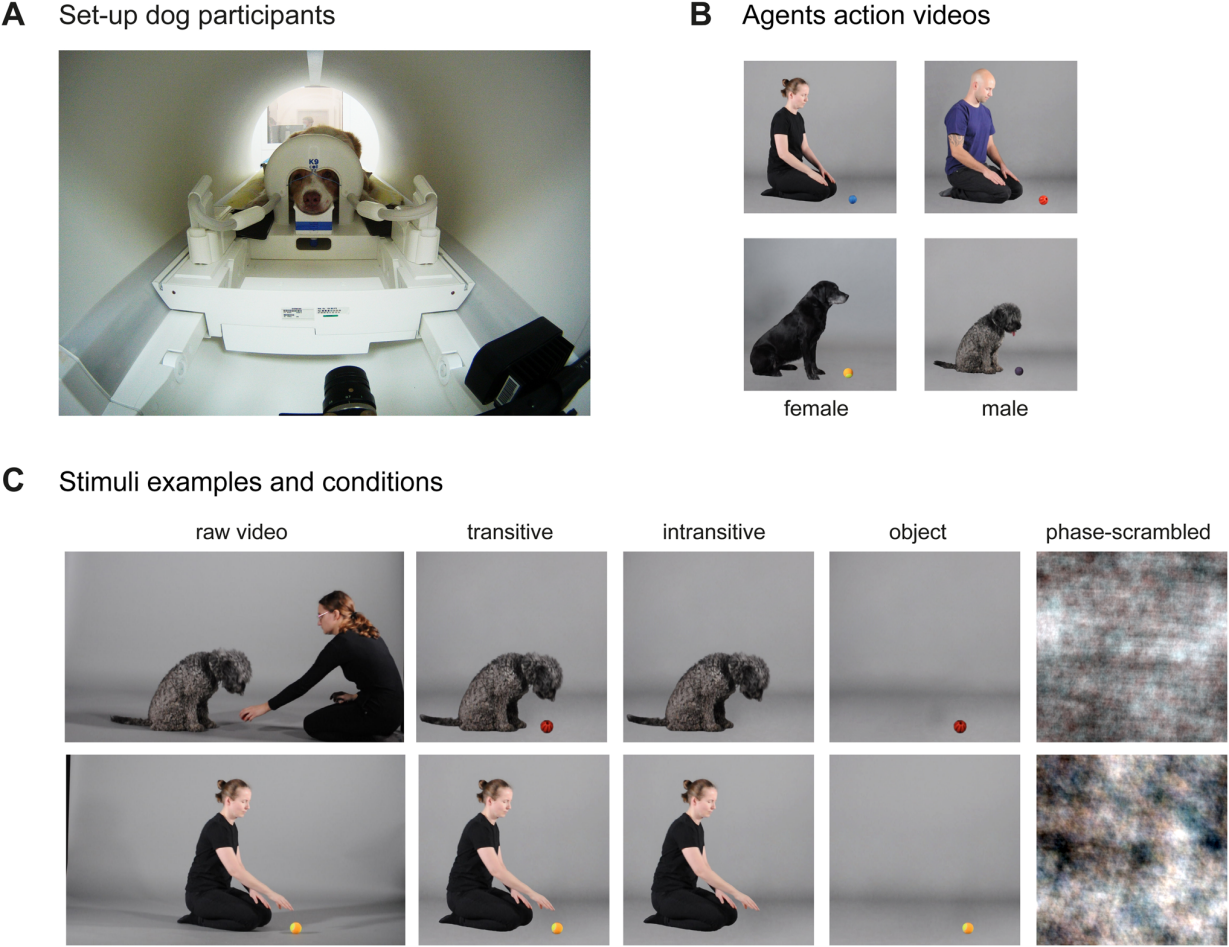
Experimental design. (A) Dogs were positioned in sphinx position with their head placed in a custom-made 16-channel (k9) head coil (Guran et al., 2023). For human participants, data were obtained with a 32-channel human head coil in a supine position (not shown). (B) All participants underwent scanning under the same conditions and using the exact same video stimuli depicting 4 unfamiliar agents (2 humans, 2 dogs) picking up different toy balls (4 videos/agent). (C) Participants saw videos of transitive and intransitive actions performed by humans and dogs and two control conditions: object and phase-scrambled motion. All conditions were derived from the same raw videos by cutting out the dog trainer if applicable (transitive), the toy (intransitive), agent (object motion), or by phase scrambling the transitive video. Stimuli were presented in a block design (12 s) with four videos per block and data acquisition was split into two 5-min task runs. All example images of the stimulus set are screenshots of material created by the authors, and we have obtained consent from individuals featured in the videos for their public dissemination.

#### 2.2.2 Agent perception localizer

In two 5-min task runs, dogs saw colour images of faces and (headless) bodies of dogs and humans, as well as inanimate objects and scrambled images (i.e., low-level visual control) presented on a grey background. As in the main task, images were presented in a block design (12 s blocks, 5 images/block) with the image composition randomized and no consecutive blocks of the same stimulus conditions (seeBoch et al., (2023b)for details on the paradigm and stimulus material).

#### 2.2.3 Procedure

Before data collection, dogs received extensive training to habituate to the scanner environment to participate without any sedation or restraints (seeKarl et al., (2020)for a detailed description of the training and data collection procedure). A trainer was also present in the scanner room to monitor and handle the dog out of its sight. We positioned the camera of an eye-tracker (Eyelink 1000 Plus, SR Research, Ontario, Canada) below the MRI screen to live-monitor participants’ attention towards the screen (i.e., whether eyes were open and directed towards the screen) and motion (see e.g.,https://osf.io/bj5quvideo of dog monitoring set-up). We stopped data acquisition if the dog missed a stimulus block due to a lack of attention, either indicated by closed eyes or a absence of visual engagement, as observed in their gaze towards the stimuli on the screen; we did not record eye-tracking data.

Dogs and humans were equipped with earplugs, and both could stop data collection anytime, either by leaving the scanner by retreating from the coil and exiting the scanner via a custom-made ramp (dogs) or pressing an alarm squeeze ball signalling to stop the scanning (humans). Human participants completed both task runs within one session with a short break in between. After each session, we evaluated the motion parameters. If overall motion for any of the three translation directions exceeded 4 mm or if scan-to-scan motion (i.e., framewise displacement to account for translational and rotational motion (Power et al., 2012,^2014^)) exceeded .5 mm in more than 50% of the scans, we re-invited the dog to repeat the task run (individual motion parameters are available on the study’s OSF data repository) or would have discarded the data from the human participant. Based on these criteria, and sufficient attentiveness evaluated by the research team based on live video observation, dogs needed on average 2.75 sessions (*SD*= 1.46) to complete both task runs successfully. Dogs had at least a 1-week break in-between data collection sessions. We did not have to replace any human data.

#### 2.2.4 Stimuli

All stimuli were created based on video recordings of a dog or human agent picking up different toy balls (see e.g.,Fig. 1B, C). To increase ecological validity and familiarity with the observed action, we asked human agents to grasp the ball with their (right) hand. We trained the dog agents to pick it up with their mouths as this would be their natural way to perform this task. We recruited two human and dog models (each a male and a female) and recorded four videos from each agent (duration: ~3 s). Videos were filmed in the same setting to ensure no differences in lighting between videos, and human and dog models were unfamiliar to the study participants. The original videos were cropped to 720 × 720 pixels, and shadows and the dog trainer were edited out to create the transitive action video. We created the intransitive action stimuli by editing out the ball from the transitive action video, the object motion control by editing out the agent, and the scrambled low-level visual control by phase-scrambling the transient action video (seeFig. 1C). Videos were edited using Adobe Photoshop and After Effects (Adobe Systems Incorporated, CA, USA).

### 2.3 MRI data acquisition

MRI data for the action observation task were obtained using a custom-made 16-channel (k9) head coil for the dog participants (Guran et al., 2023) (seeFig. 1A) and a 32-channel head coil for human participants, used in a 3T Siemens Skyra MR-system (Siemens Medical, Erlangen, Germany). For three dogs, we used structural scans which had been previously acquired with a 15-channel human knee coil due to unavailability of the dogs to acquire a new structural scan. However, structural sequences for both coils were the same and qualitative comparisons showed that image registration worked equally well with structural scans acquired from both coils. For the agent localizer, data from*n*= 15 dogs (i.e., from the original publication of the localizer paradigm (Boch et al., 2023a)) were acquired with the human knee coil and from*n*= 13 dogs with the k9 head coil.

For all dog functional scans, we used a 2-fold multiband (MB) accelerated echo planar imaging (EPI) sequence for the dog functional scans with the following parameters: voxel size = 1.5 × 1.5 × 2 mm^3^, repetition time (TR)/echo time (TE) = 1,000/38 ms, field of view (FoV) = 144 × 144 × 58 mm^3^, flip angle = 61°, 20% gap, 24 axial slices (interleaved acquisition, descending order) with a posterior-to-anterior phase-encoding direction. Individual numbers of volumes per action observation task run vary slightly because task acquisition was stopped manually (mean = 324 volumes;*SD*= 10). We also acquired field map scans; however, due to a technical error, the large majority of the scans could not be used, and we processed the dog data without it. Voxel size for all structural scans was .7 mm isotropic (TR/TE = 2,100/3.13 ms, FoV = 230 × 230 × 165 mm^3^).

We acquired human functional scans (each run: mean = 272 volumes*SD*= 2) with a 4-fold MB accelerated EPI sequence: voxel size = 2 mm isotropic, TR/TE = 1,200/34 ms, FoV = 192 × 192 × 124.8 mm^3^, flip angle = 66°, 20% gap, and 52 axial slices coplanar to the connecting line between anterior and posterior commissure (interleaved acquisition, ascending order). In the same orientation as functional scans, we obtained additional field map scans to correct for magnetic field inhomogeneities using a double echo gradient echo sequence with a voxel size of 1.72 x 1.72 x 3.85 mm^3^, TR/TE1/TE2 = 400/4.92/7.38 ms, FoV = 220 × 220 × 138 mm^3^, flip angle = 60° containing 36 axial slices. Voxel size for structural scans was .8 mm isotropic with TR/TE = 2,300/2.43 ms and FoV = 256 × 256 × 166 mm^3^.

### 2.4 Data preprocessing

We preprocessed and analysed imaging data of both species using SPM12 (https://www.fil.ion.ucl.ac.uk/spm/software/spm12/), Matlab 2020b (MathWorks Inc., MA, USA), and R 4.3.0 (R Core Team, 2023). For the dogs, after slice timing correction (reference: middle slice) and realignment, we manually reoriented the functional and structural images and set the origin at the anterior commissure using the SPM*Display*function to match the orientation of the dog template (Nitzsche et al., 2019). Next, we skull stripped the structural images using individual binary brain masks created with itk-SNAP (Yushkevich et al., 2006), co-registered the brain-extracted structural images to the mean functional images, and segmented them. We then normalized and resliced (1.5 mm isotropic) all imaging data to the breed-averaged dog template space (Nitzsche et al., 2019) and smoothed the data with a 3-dimensional Gaussian Kernel (full-width-at-half-maximum, FWHM; 3 mm; i.e., twice the raw within-plane voxel resolution). Structural scans were acquired in a separate session to allow for longer functional MRI task runs. Since we train the dogs to maintain a consistent head position and manually reorient both structural and functional scans to match the dog template space before image registration, variations in head position between sessions are minimal. Therefore, registration accuracy is unlikely to be affected. Nonetheless, we carefully checked image registration after co-registration and normalization for each dog, using significant landmarks such as the ventricles, corpus callosum, or brain borders, and found no instances of poor registration.

For the human data, we realigned and unwarped functional images after slice time correction (reference: middle slice) using the individual field maps. Individual structural images were then co-registered to the mean functional images and segmented. Next, we normalized and resliced (1.5 mm isotropic) the imaging data and applied spatial smoothing with a 3-dimensional Gaussian Kernel (FWHM, 4 mm; i.e., twice the raw voxel resolution).

Next, we calculated individual scan-to-scan motion (i.e., framewise displacement, FD) to account for translational and rotational motion in both species. For each scan exceeding the a priori set FD threshold of .5 mm, we added a motion regressor to the first-level general linear models (Power et al., 2012, GLMs; motion scrubbing 2014). On average, 5.7% of the dog scans (run 1: mean FD = .2 mm, 90^th^percentile = .35 mm; run 2: mean FD = .21 mm, 90^th^percentile = .32 mm) and 1.5% of the human scans (run 1: mean FD = .18 mm, 90^th^percentile = .23 mm; run 2: mean FD = .18 mm, 90^th^percentile = .25 mm) exceeded the threshold in each run.

### 2.5 Overview of the main analyses and rationale

**Fig. 2.**
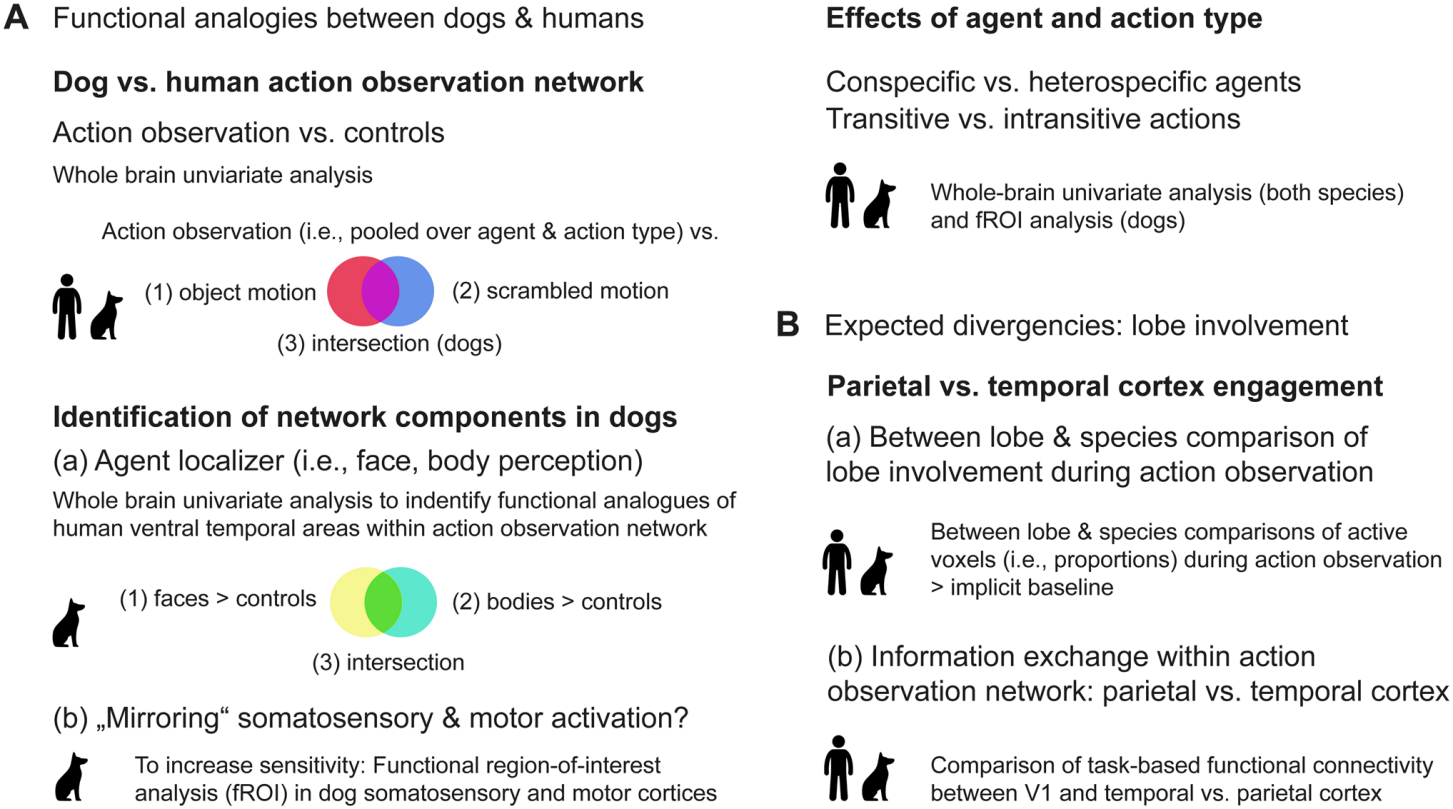
Graphical overview of the main analysis steps. The first part (A) focused on exploring the dog action observation network and identified functionally analogous components to the human action observation network. (B) The second part investigated hypothesized divergencies regarding the relative involvement of the temporal and parietal cortex in dogs and humans during action observation.

The aim of the first part of our analyses was to localize the dog action observation network for the first time (Fig. 2A) and, based on that, to identify functional analogies between the dog and human action observation networks by applying whole-brain univariate and region-of-interest (ROI) analyses. To this end, we applied the following analysis steps.

First, we identified the species’ action observation networks by comparing activation for both agents and action types (i.e., pooled activation) to the implicit baseline and the two control conditions and calculated the intersection between the two contrasts. Second, we investigated the components of the dog action observation network. To detect face- and body-sensitive areas (i.e., agent areas) in the dog temporal lobe, we used a functional localizer task, which allowed us to identify the functional analogues of the human ventral visual pathway (Haxby et al., 2000;Ungerleider & Haxby, 1994) within the dog action observation network. This also allowed us to test our hypothesis of temporal cortex engagement beyond the agent areas in dogs, functionally analogous to humans (i.e., lateral temporal or third visual pathway (Pitcher & Ungerleider, 2021;Wurm & Caramazza, 2022)). Due to anticipated low tSNR in parts of the sensorimotor cortices, we also conducted a univariate ROI analysis in addition to the whole-brain analysis in dogs to test our hypothesis of somatosensory and (pre-) motor activation in response to action observation as this approach results in higher sensitivity. Third, we tested our hypothesis of comparable activation for transitive versus intransitive actions due to the species’ imitation skill, and we explored how the human and dog action observation network responded to observing actions performed by conspecifics versus heterospecifics.

The second part of our analysis then focused on between-species divergencies (Fig. 2B). Here, we tested our key hypothesis of functional divergencies in temporal versus parietal cortex engagement in the two species. Based on prior non-human primate and human research and the species’ differential relative lobe expansion, we specifically expected a stronger temporal than parietal cortex engagement during action observation in dogs but a more balanced involvement of both lobes in humans. We tested this hypothesis by implementing two analysis approaches. First, we conducted within- and cross-species comparisons of the extent of active voxels in each species’ temporal and parietal cortex during action observation. Second, we compared task-based functional connectivity with the primary visual cortex and the temporal versus parietal cortex. This also allowed us to explore information exchange within the species’ action observation networks.

### 2.6 Univariate analysis

#### 2.6.1 Action observation task

##### 2.6.1.1 First-level analysis

First-level analyses were conducted using a GLM approach implemented in SPM12 (https://www.fil.ion.ucl.ac.uk/spm/software/spm12/). We defined four task regressors for our main conditions of interest: transitive and intransitive actions of each species (i.e., dog/human × transitive/intransitive action) and two for the control conditions (i.e., object motion and scrambled). All blocks were estimated as a boxcar function time-locked to the onset of each block with a duration of 12 s and convolved with a tailored dog haemodynamic response function (HRF;Boch et al., 2021) or the standard human HRF implemented in SPM12. In addition, we added the six motion regressors retrieved from image realignment and the individual framewise displacement regressors as nuisance regressors. We applied a temporal high-pass filter with a cut-off at 128 s. Due to signal distortions caused by dogs’ large sinuses potentially affecting signal in the dog frontal lobes and, therefore, partly the sensory–motor cortices (seeFig. 3A, B), we applied implicit masking to exclude voxels with a mean value lower than 80% of the global signal. We also calculated individual whole-brain temporal signal-to-noise ratio (tSNR) maps$\left(tSNR=\;\frac{voxelwise\;mean\;\left(time\;series\right)}{voxelwise\;SD\;\left(time\;series\right)}\right)$to measure where large sinuses might have affected individual brain signal in dogs.

**Fig. 3.**
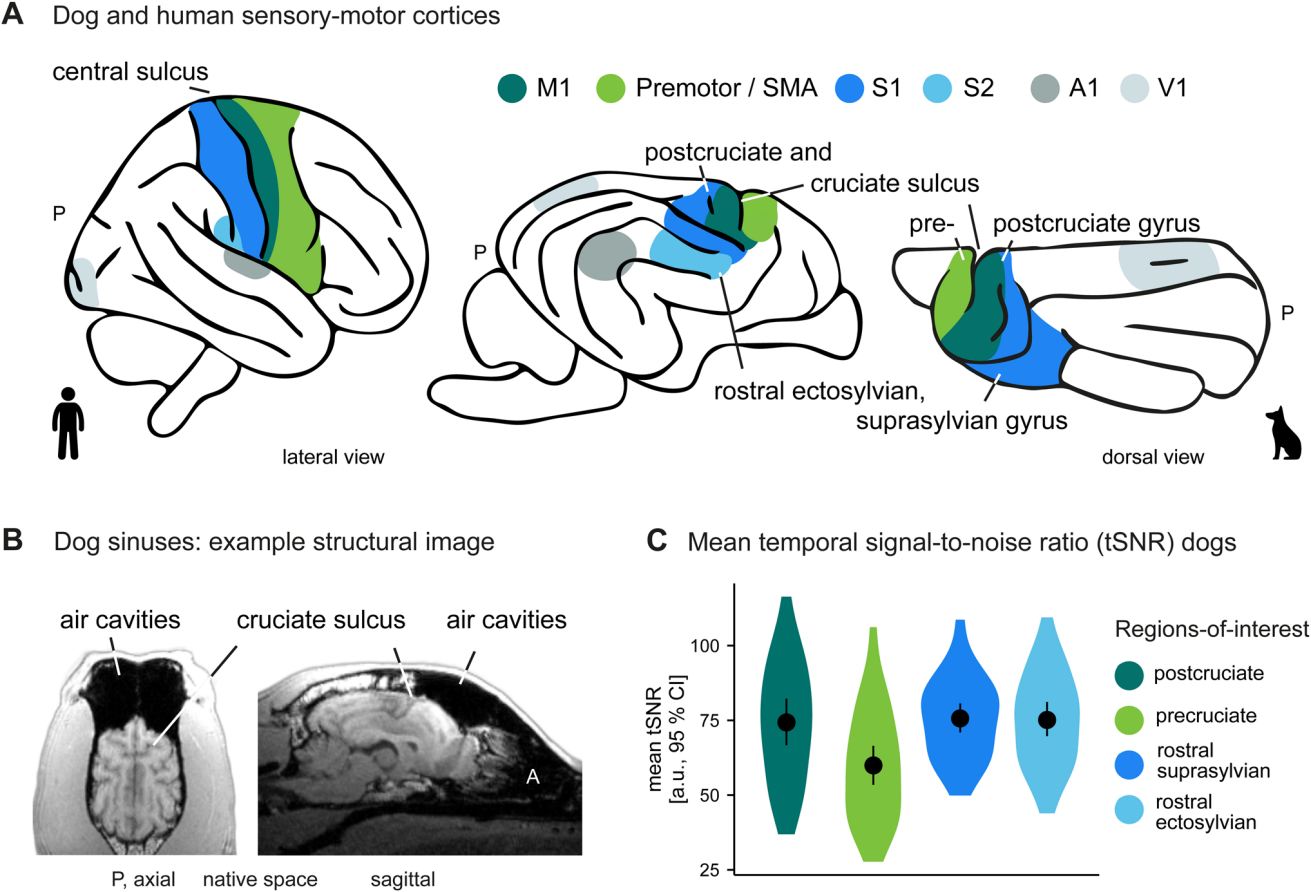
Decreased temporal signal-to-noise (tSNR) ratio in dog premotor and secondary motor cortices housed in precruciate gyrus. (A) For visual comparison and guidance, we created a schematic figure of the human and dog sensory–motor cortices along with the primary visual (V1) and auditory (A1) cortices; see alsoFigure 4for a schematic overview of all dog gyri and sulci mentioned in the study andBoch et al. (2024)for a review of dog sensory areas. The central sulcus marks the border between primary somatosensory (S1) and motor (M1) cortices in humans. The dog analogue of the human central sulcus is the less pronounced postcruciate sulcus, indicating the transition between M1 and S1 cortex (see e.g.,Kreiner, 1964c;Pinto Hamuy et al., 1956;Tanaka et al., 1981). S1 further extends to the dog rostral suprasylvian gyrus, and S2 is located in the rostral ectosylvian gyrus ventral to S1 (Pinto Hamuy et al., 1956). The cruciate sulcus marks the transition from M1 to premotor and supplementary motor (SMA) cortex housed in the precruciate gyrus (Adrian, 1941;Gorska, 1974;Tanaka et al., 1981). (B) As presented in the example structural image from one of the dogs in our study sample (Border Collie Australian Shepherd mix), dogs have large sinuses located anterior–superior to the frontal lobes, heavily affecting the signal and resulting in signal drop out in these areas when using T2*-weighted (fMRI) scans. This includes the precruciate gyrus in large parts (i.e., dog premotor and supplementary motor areas). (C) Temporal signal-to-noise ratio (tSNR) measures were considerably lower in the precruciate gyrus (range: 27.71–106.16; 90^th^percentile: 84.36) compared with the other gyri housing somatosensory or motor areas. The violin plots show the group mean tSNR measured in arbitrary units (a.u.) with error bars indicating the 95% confidence interval (CI) and kernel density plots. Individual whole-brain tSNR maps are provided on the study’s OSF data repository. P, posterior. The dog and human icons in A were purchased fromhttps://thenounproject.com(royalty-free license).

Finally, we computed contrasts for all six task regressors, an action observation contrast averaging the activation for transitive and intransitive actions of both species (i.e., all conditions showing agent performing an action > implicit visual baseline) and a task-activation contrasts (i.e., all task conditions > implicit visual baseline).

##### 2.6.1.2 Whole-brain group comparisons

First, we conducted a one-sample*t*-test for the action observation contrast (i.e., all action conditions > implicit visual baseline) for a first exploration of the action observation network and one sample*t*-tests for each condition of interest. We then implemented two paired-sample*t*-tests to compare the activation elicited by action observation to (1) the low-level visual stimulation control (i.e., all action conditions > phase-scrambled motion) and (2) the activation elicited by object motion (i.e., all action conditions > object motion condition). To investigate whether the dog and human action networks respond to conspecific and heterospecific actions and to test our hypothesis of no pronounced differences in activation for transitive and intransitive actions due to the species imitation skills, we calculated a within-subjects full factorial model using the flexible factorial framework in SPM12 with the factors*action*(transitive, intransitive) and*agent*(dog, human) to test for main effects of action and agent, and an action × agent interaction.

The dog sample had a broader range of ages than the human sample, included various breeds with different brain sizes, and many dogs had more exposure to the stimuli than humans because some task runs had to be repeated due to excessive motion. To assess the potential effects of these factors on the observed activation during action observation in dogs, we conducted a series of secondary analyses in dogs. First, to explore whether repeated stimuli exposure affected the observed activation in dogs, we conducted multiple regression analyses for the contrasts transitive action observation > implicit baseline and intransitive action observation > implicit baseline and added the number of sessions to complete both task runs as a covariate. We used the contrasts from the second task run, where the effects of stimulus exposure, if present, would be expected to be most pronounced. Previous research indicates significant variation in brain structure across dog breeds, with systematic differences in grey matter volume correlating with behaviour and brain shape (i.e., neurocephalic index;Hecht et al., 2019,^2021^). While our sample size and breed variance did not allow us to test for breed-specific differences, we investigated the effects of individual grey matter volume, brain shape, and age. Based on the findings fromHecht et al. (2019,^2021^), we focused on grey matter volume instead of whole brain size. We calculated grey matter volume by counting the number of voxels within the individual grey matter segmentations in native space and multiplying it by the volume of one voxel (.7 mm × .7 mm × .7 mm = .343 mm^3^). For the neurocephalic index, we used itk-SNAP (Yushkevich et al., 2006) to identify the coordinates of the maximal distances along the anterior–posterior and left–right axes in each dog’s structural brain scan. We then computed the Euclidean distances to determine brain width and length and calculated the neurocephalic index as the ratio of brain width to length multiplied by 100. Finally, to assess the effects of grey matter volume, brain shape, and age (in years) on activation during action observation, we conducted multiple regression analyses for the contrasts transitive action observation > implicit baseline and intransitive action observation > implicit baseline, in which we added volume, brain shape, and age as covariates.

For all whole-brain group analyses, we determined significance by applying cluster-level inference with a cluster-defining threshold of*p*< .005/.001 (dogs/humans) and a cluster probability of*p*< .05 family-wise error (FWE) corrected for multiple comparisons. To account for the smaller sample size, we made an a priori decision to choose a lower cluster-defining threshold for the dog data. This threshold has been commonly used in the field of dog fMRI (e.g.,Boch et al., 2023b;Bunford et al., 2020;Karl et al., 2021), suggesting its effectiveness in identifying relevant brain responses while at the same time increasing comparability to and integration of the present data with prior findings data. However, to allow for between-species comparisons using the identical statistical threshold, we also report results for the main contrasts of interest (action observation > implicit baseline / scrambled motion / object motion) and the full factorial model (agent, action type) using the same threshold as for the human data. We derived the cluster extent (i.e., the minimum spatial extent to be labelled significant) using the SPM extension “CorrClusTh.m” (Nichols & Wilke, 2012). Anatomical labelling of activation peaks and clusters of all reported results refers to the dog brain atlas from Czeibert and colleagues (Czeibert et al., 2019) normalized to the stereotaxic breed-averaged template space (Nitzsche et al., 2019), and the Harvard–Oxford human brain atlas (Desikan et al., 2006), and was performed using the Python software AtlasReader (Notter et al., 2019).

##### 2.6.1.3 Univariate region-of-interest (ROI) analysis of dog sensorimotor cortex

We hypothesized somatosensory and (pre-) motor areas to be part of the action observation network in dogs, functionally analogous to humans. Due to the anticipated lower tSNR in this area in dogs, we conducted an ROI analysis to test this hypothesis with higher sensitivity. Constrained masks for somatosensory and motor cortices of the dog brain do not exist, and knowledge about the exact locations, especially of premotor and secondary motor cortices, is limited. However, it is known which gyri house sensorimotor cortices. The premotor cortex and supplementary motor area (SMA) are located in the precruciate gyrus (Gorska, 1974;Kreiner, 1964c;Tanaka et al., 1981). The motor cortex is housed in the anterior portion of the postcruciate gyrus, bordering the primary somatosensory cortex (S1) posteriorly at the postcruciate sulcus and ventrally at the rostral suprasylvian sulcus (Gorska, 1974;Kreiner, 1964c;Tanaka et al., 1981). S1 encompasses the posterior postcruciate gyrus and the rostral suprasylvian gyrus, and the secondary somatosensory cortex (S2) is housed in the rostral ectosylvian sulcus (Adrian, 1941;Pinto Hamuy et al., 1956; see alsoFig. 3AandBoch et al., 2024, for a detailed review). We, therefore, defined four ROIs using the following publicly available gyri masks (Czeibert et al., 2019): precruciate gyrus (“premotor and SMA”), postcruciate gyrus (“M1/S1” or “somatomotor”), rostral suprasylvian gyrus (“rostral suprasylvian S1”), rostral ectosylvian gyrus (“S2”). To constrain the large anatomical masks, we created a binary mask of the group task-based activation (i.e., all conditions > implicit visual baseline) liberally thresholded at*p*= .05 uncorrected and intersected the functional mask and each of the anatomical gyri masks. Next, using the REX toolbox (Duff et al., 2007), we extracted parameter estimates for each condition of interest (i.e., pooled actions contrast, dog/human transitive/intransitive action) and the controls (i.e., object and scrambled motion) from the sensorimotor ROI masks.

First, to test whether areas of the sensorimotor cortex respond stronger to action observation compared with controls, we employed linear mixed models (LMMs) for each of the four ROIs using the R packages*lme4*(Bates et al., 2015) and*afex*(Singmann et al., 2020). We defined activation levels (i.e., parameter estimates) as the dependent variable,*condition*(levels: actions, object motion, phase-scrambled motions) as predictor, and added per-subject random intercepts. To investigate potential differences in activation due to the observed agent or action type, we conducted LMMs for each ROI, with activation levels as the dependent variable and*agent*(levels: dog, human) and*action*(levels: transitive, intransitive) as predictors (i.e., 2 × 2 within-subjects design) and added by-subject random intercepts. We applied false-discovery rate (FDR) control to correct*p*-values for group comparisons investigating the same research questions (e.g., greater activation for action observation compared with controls) and for planned post hoc comparison.

After reviewing the results, we conducted an additional exploratory analysis by re-examining the precruciate activation levels in a sub-sample of the seven dogs with the top 25% of temporal signal-to-noise ratio (tSNR) values in this area (seeFig. 3C).

##### 2.6.1.4 Cross-species comparison of parietal and temporal cortex involvement

As outlined above, we predicted stronger temporal than parietal cortex activation in dogs and significantly less activation in the dog compared with the human parietal cortex during action observation. To quantitatively test the potential cross-species differences in parietal and temporal activation, we measured the individual percentages of active voxels within each lobe during action observation (i.e.,$\left(\;\frac{number\;of\;active\;voxels}{total\;number\;of\;voxels\;within\;masks}\;\right)\times 100$). Specifically, we focused on the higher-order visual and multisensory cortex and excluded somatosensory (described also above and inFig. 3A) and auditory areas of both species from the lobe masks and subsequent analysis (see alsoFig. 4for main gyri of the dog brain and the brain masks).

**Fig. 4.**
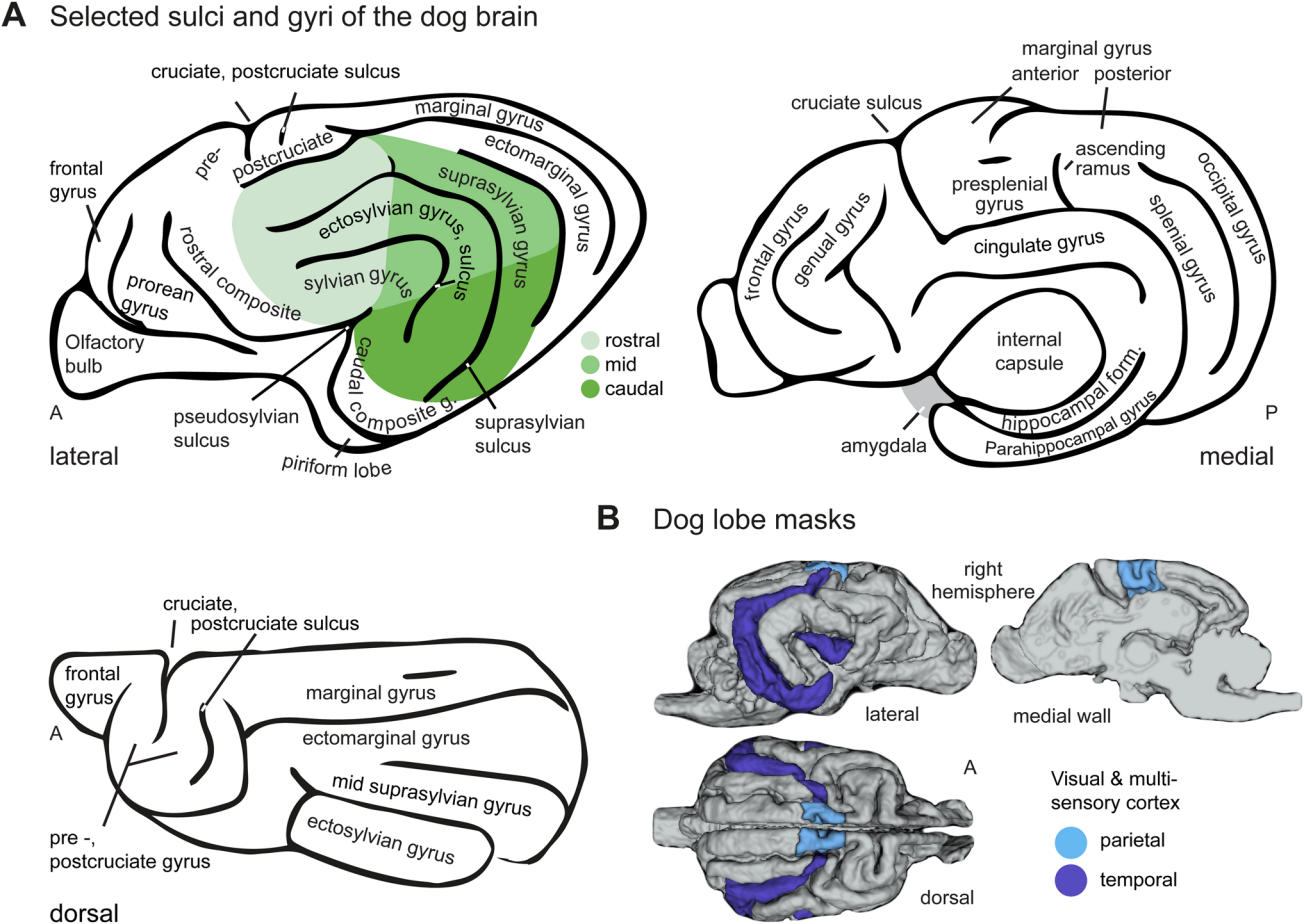
Selected gyri and sulci of the dog brain and temporal and parietal cortex masks. (A) Lateral, medial, and dorsal view of the dog brain (schematic drawings), including relevant gyri and sulci for the present study, accompanied by major anatomical landmarks for visual guidance. (B) The lobe masks included higher-order visual and multisensory areas of dogs’ parietal (rostral marginal and ectomarginal, and presplenial gyrus) and temporal (mid and caudal suprasylvian, rostral sylvian and caudal composite gyrus) cortices. Known auditory regions in the temporal lobe (i.e., caudal and mid ectosylvian gyrus and caudal sylvian gyrus; see e.g.,Kosmal, 2000,^2004^;Tunturi, 1950) and somatosensory regions (parietal rostral suprasylvian gyrus and postcruciate gyrus; see e.g.,Adrian, 1941;Pinto Hamuy et al., 1956;Fig. 3) were excluded from the masks (see alsoBoch et al., 2024for review of dog sensory areas). A, anterior; P, posterior; g., gyrus.

For the dogs, we created the parietal cortex mask by combining bilateral anatomical masks from Czeibert and colleagues (Czeibert et al., 2019) of the anterior portions of the marginal and ectomarginal gyrus with the ascending ramus indicating the posterior border and the presplenial gyrus (Johnson et al., 2020;Kreiner, 1964a). We did not include the postcruciate gyrus because it houses primary somatosensory and motor areas (Gorska, 1974;Kreiner, 1964c;Tanaka et al., 1981).

Dog temporal cortex definitions vary regarding the inclusion or exclusion of the mid and caudal suprasylvian gyrus (Johnson et al., 2020;Kreiner, 1964b;Nitzsche et al., 2019). Considering the functional convergence between the mid and caudal suprasylvian agent areas and the human inferior temporal cortex (Boch et al., 2023b;Bunford et al., 2020; and seeBoch et al., 2024for review), we included both gyri in the dog temporal lobe mask together with the multisensory caudal composite and rostral sylvian gyri (Kosmal et al., 2004). The mid and caudal parts of the ectosylvian gyrus were not included in the mask because they house primary and higher-order auditory regions (Kosmal, 2000;Kosmal et al., 2004), and the rostral part houses S2 (Adrian, 1941;Pinto Hamuy et al., 1956; seeFig. 3). The temporal cortex mask covered 1,676 voxels, and the parietal cortex mask 301 voxels.

We created the human lobe masks using anatomical masks from the Harvard–Oxford brain atlas (Desikan et al., 2006). The bilateral temporal mask includes the temporal pole, all middle and inferior temporal gyrus masks and the temporal fusiform cortex. For the parietal cortex mask, we combined bilateral masks of the superior parietal lobule, supramarginal gyrus (anterior and posterior division), angular gyrus, and precuneus cortex. The temporal cortex mask covered 30,792 voxels, and the parietal cortex mask 22,514 voxels.

As for the group analysis, we determined significantly active voxels by thresholding the individual contrast maps for the action observation contrast (i.e., all conditions displaying an agent performing an action > implicit baseline) using cluster-level inference. We also conducted secondary analyses with more liberal thresholds to ensure cross-species comparisons were not biased due to too conservative thresholds. First, we applied an uncorrected threshold of*p*< .005/.001 for dogs/humans to determine significant voxels. Second, following procedures established in comparative primate neuroimaging research (Hecht, Murphy, et al., 2013;Rilling et al., 2007), we defined the most active voxels (i.e., highest positive beta-values) as significant. We used the top 5% voxels as the threshold but also calculated parietal and temporal cortex involvement for percentage thresholds ranging from 1% to 100% in steps of 5% for a further visual inspection.

For group (cross-species) comparisons, we employed an LMM with proportions (i.e., individual percentages of active voxels) as the dependent variable and*lobe*(levels: parietal, temporal; within-subject) and*sample*(levels: dog, human participants; between-subject) as predictors and added by-subject random intercepts.*p*-Values for planned post hoc comparisons were FDR controlled.

#### 2.6.2 Agent localizer

We employed a functional localizer task to locate face- and body-sensitive regions (i.e., agent regions) in our sample in order to identify the functional analogues of the human ventral visual pathway (Ungerleider & Haxby, 1994) within the dog’s action observation network.

##### 2.6.2.1 First-level analysis

As described above, first-level analyses were conducted using a GLM approach implemented in SPM12. We defined four task regressors for the main conditions of interest: faces and bodies of each species (i.e., dog/human faces/bodies) and two for the control conditions (i.e., inanimate objects and scrambled). We then computed averaged contrasts for faces and bodies (i.e., pooled activation for dog and human stimuli), for dogs and humans (i.e., pooled for faces and bodies), and for inanimate objects (all conditions > scrambled control).

##### 2.6.2.2 Group comparison

To localize the face- and body-sensitive areas, we conducted a whole-brain one-way repeated measures analysis of variance (ANOVA; levels: faces, bodies, inanimate objects; all conditions > scrambled controls) and a second ANOVA to investigate potential differences due to species depicted with the factors*agent*(dog, human) and*section*(face, body; all conditions > scrambled controls). Both ANOVAs were implemented using the flexible factorial framework in SPM12.

### 2.7 Task-based functional connectivity analysis

As outlined above, we expected stronger temporal than parietal cortex engagement during action observation in dogs, but a more balanced involvement of the two lobes in humans. In addition to the comparison of activation extent, we also aimed to compare the strength of information exchange (i.e., task-based functional connectivity) with V1 between the parietal and temporal cortex in the two species.

#### 2.7.1 Action observation task

##### 2.7.1.1 First-level analyses

To investigate whether action observation led to differences in functional connectivity between the primary visual cortex (i.e., seed region) and the temporal and parietal cortices in both species, we used generalized psychophysiological interaction (gPPI) analyses (McLaren et al., 2012). For the dogs, due to the unavailability of an anatomical mask, we created a primary visual cortex (V1) sphere (x = 1, y = -29, z = 16, 4 mm) using coordinates from a visual localizer task (Boch et al., 2021). We built the human V1 seed region by combining the supra- and intracalcarine cortex masks from the Harvard–Oxford brain atlas (Desikan et al., 2006). From the respective V1 masks, we extracted the first eigenvariate of the individual functional time courses of the dog and human participants, adjusted the functional time courses for average activation using an F-contrast, and deconvolved them to estimate the neural activity in the seed region (i.e., physiological factor). We then multiplied the neural activation estimate with a boxcar function time linked to the onset of each block (i.e., psychological factor) convolved with the species-specific HRF models (Boch et al., 2021). This resulted in one psychophysiological interaction regressor per condition of interest. The interaction regressors were added to the first-level design matrix, and the GLMs were estimated. Using the REX toolbox (Duff et al., 2007), we then extracted mean functional connectivity estimates between the seed region (i.e., V1) and agent- and action-sensitive areas in both species (i.e., target regions) for each condition of interest (i.e., pooled actions contrast, dog/human transitive/intransitive action) and the controls (i.e., object and scrambled motion).

##### 2.7.1.2 Group comparisons

On the group level, we investigated whether action observation led to greater functional connectivity between V1 and agent- and action-sensitive areas than the control conditions and whether task-based functional connectivity between V1 and the temporal versus parietal lobe differed in dogs and humans (see alsoSection 2.7.1.3below). First, we aggregated the functional connectivity estimates across all anatomical masks of the same lobe and employed an LMM for each species with the aggregated functional connectivity as the dependent variable,*lobe*(levels: parietal, temporal; within-subject) and*condition*(levels: actions, object motion, scrambled motion) as predictors and random intercepts as well as random slopes for*lobe*and*condition*. This corresponds to the maximal random effects structure possible with this design, as recommended byBarr et al. (2013). The factor*condition*was contrast coded using Helmert coding (contrast 1: actions – (object motion + scrambled motion) / 2; contrast 2: scrambled motion – object motion) to directly test our contrast of interest (i.e., contrast 1). Finally, we investigated task-based functional connectivity changes in each anatomical area separately by setting an LMM with functional connectivity as the dependent variable for each anatomical area,*condition*(levels: actions, object motion, scrambled motion) as the predictor, and we added by-subject random intercepts.*p*-Values for planned post hoc comparisons and analyses investigating the same research question were FDR controlled.

##### 2.7.1.3 Regions-of-interest task-based functional connectivity

The anatomical areas included in the parietal and temporal lobe masks described above (Section 2.6.1.4) served as the target regions for the dogs. For the human participants, we used anatomical masks (retrieved again from the Harvard–Oxford brain atlas;Desikan et al., 2006) of the known temporal and parietal core nodes of the action observation network. This included the superior parietal lobule, the supramarginal gyrus in the human parietal lobe, and the posterior fusiform cortex, and the posterior superior temporal sulcus (pSTS) area in the temporal lobe. Due to the lack of a pSTS mask in volumetric space, we created and subsequently combined left and right hemisphere spheres based on coordinates from previous work investigating the functional organization of the STS (Schobert et al., 2018) (sphere radius: 10 mm; left centre x = -50, y = -48, z = 15; right: x = 50, y = -47, z = 13). Finally, we removed all voxels overlapping with the pSTS mask from the supramarginal gyrus mask.

Our rationale to define the target regions for the main functional connectivity analysis anatomically was because the univariate activation analysis did not reveal any action-sensitive areas in the dog parietal lobe, and we did not want the lobe functional connectivity comparison to be biased by comparing more constrained functionally defined target areas with anatomical masks. However, in a secondary analysis, we also investigated functional connectivity between V1 and functionally defined action- and agent-sensitive areas in the dog temporal lobe based on the univariate activation results (i.e., face- and body-sensitive areas localized with the agent localizer and cluster resulting from the overlap actions > object motion∩actions > scrambled motion) to assess connectivity of the areas identified as part of the dog action observation network.

#### 2.7.2 Agent localizer

The ventral visual pathway expands from the primary visual cortex to the inferior temporal cortex of humans (Haxby et al., 2000;Ungerleider & Haxby, 1994), representing one component of the human action observation network. The univariate analysis of the functional (agent) localizer served to detect the functional analogues of this pathway within the dog action observation network. In this secondary analysis, we further examined whether the localized dog face- and body-sensitive areas are functional analogues of the human ventral visual pathway (Haxby et al., 2000;Ungerleider & Haxby, 1994) by investigating whether these areas exchange information with V1 during the perception of static faces and bodies compared with controls.

##### 2.7.2.1 First-level analysis

We employed gPPI analyses as described above in detail using the same V1 sphere. Using the REX toolbox (Duff et al., 2007), we then extracted mean functional connectivity between the seed region and agent-sensitive areas in dogs (i.e., target regions) for each condition of interest (i.e., faces, bodies, dog/human faces/bodies) and the controls (i.e., inanimate objects, scrambled images). Target regions in the mid and caudal suprasylvian gyrus were defined based on the univariate results from the contrasts faces > controls, bodies > controls, bodies > faces.

##### 2.7.2.2 Group comparison

To investigate task-based functional connectivity changes between V1 and the agent areas, we set up one LMM for each region with functional connectivity as the dependent variable. We defined*condition*(levels: faces, bodies, inanimate objects, scrambled images) as the predictor and added by-subject random intercepts.*p*-Values for planned post hoc comparisons and analyses investigating the same research question were FDR controlled.

## 3 Results

### 3.1 Functionally convergent temporal and somatosensory components of the dog and human action observation network

First, to localize the dog and human action observation network, we investigated activation in response to the observation of transitive (i.e., goal-directed) and intransitive (i.e., goal-absent) actions of both species (i.e., average activation of all action conditions) compared with the implicit visual baseline and the object and scrambled motion controls.

**Fig. 5.**
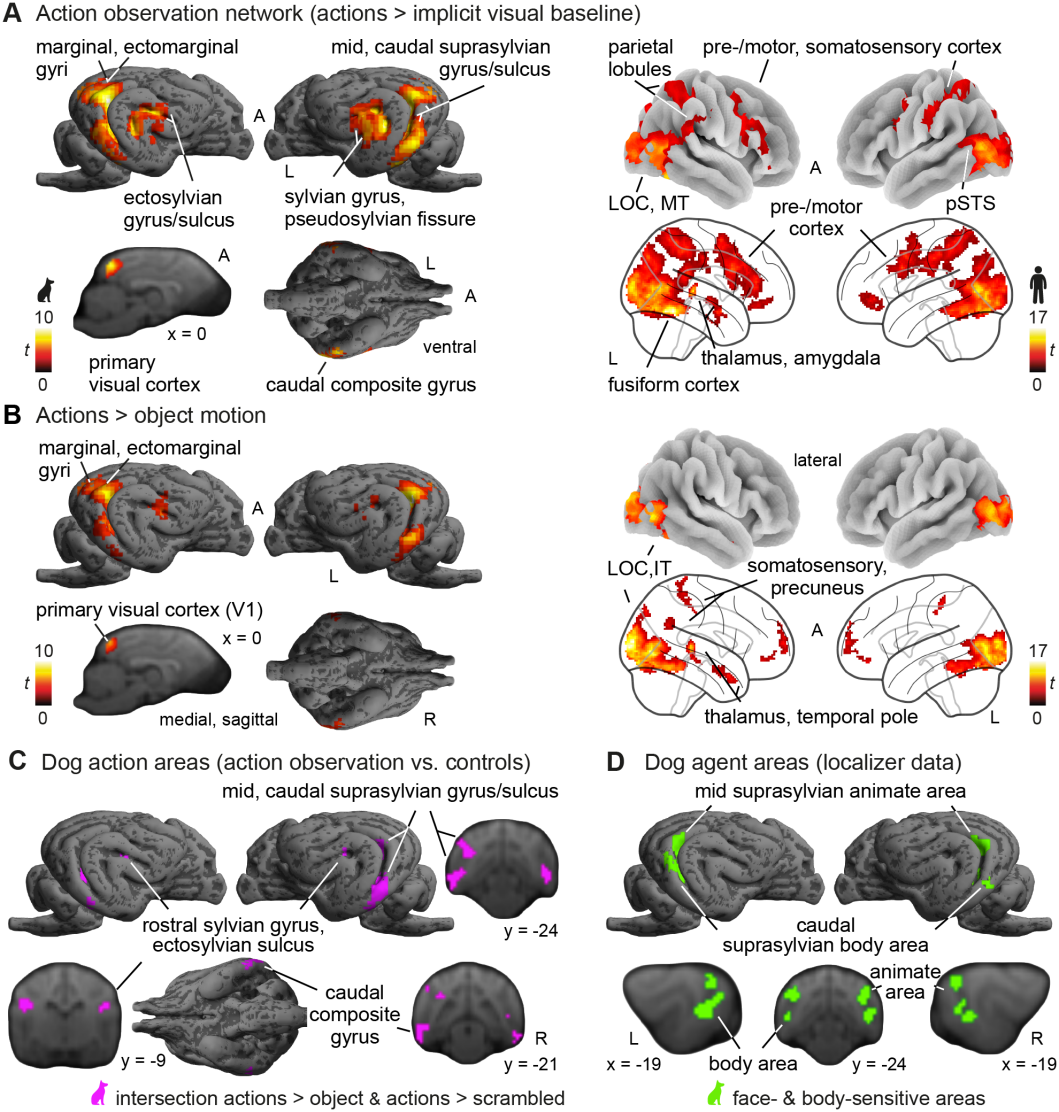
Dog and human action observation networks. (A) Action observation (i.e., pooled activation for transitive and intransitive actions of dogs and humans) compared to baseline led to functionally analogous activation in dog and human occipito-temporal and somatosensory cortex, but parietal and (pre-)motor activation was only observed in humans (Supplementary Tables S1-S2). (B) Action observation compared to object motion led to occipito-temporal activation in both species, and parietal and somatosensory activation in humans. (C) Action observation compared to object and scrambled motion (i.e., purple-coloured clusters represent the overlap of both contrasts; seeSupplementary Figure S1for each) revealed activation in the dog caudal composite and rostral sylvian gyrus including the ectosylvian sulcus (henceforth referred to as rostral sylvian area), and the mid and caudal suprasylvian gyrus and sulcus. (D) The latter two largely overlap with the mid suprasylvian animate area, which is sensitive to faces and bodies, and the caudal suprasylvian body area, which responds more strongly to bodies than faces or visual controls in the agent localizer task. Green-coloured clusters represent the overlap between the contrasts faces/bodies > controls and bodies > faces (seeSupplementary Figure S6for each contrast). Results are*p* < .05 FWE-corrected at cluster-level using a cluster-defining threshold of*p* < .005/.001 for dogs/humans. Anatomical nomenclature for all figures refers to the dog brain atlas from (Czeibert et al., 2019) normalized to the stereotaxic breed-averaged template space (Nitzsche et al., 2019) and the Harvard-Oxford human brain atlas (Desikan et al., 2006). MT, middle temporal visual area; A, anterior; R, right; L, left;*t*,*t*-values. Icons were purchased fromthenounproject.com(royalty-free license).

Action observation, compared with the implicit visual baseline, led to activation in occipitotemporal and somatosensory cortex in dogs and humans alike (seeFig 5A;Supplementary Tables S1 and S2). In dogs, occipitotemporal clusters expanded across primary (marginal gyrus) and extrastriate (splenial, ectomarginal gyrus) visual cortex, and further areas in higher-order association cortices, including the mid and caudal suprasylvian and the sylvian and ventral caudal composite gyrus (seeFig. 4Afor overview of the main gyri and sulci of the dog brain). We also found activation in the secondary somatosensory cortex (rostral ectosylvian gyrus) but no evidence for the involvement of premotor regions in the precruciate gyrus. Due to anticipated signal loss in this area caused by dogs’ large nasal cavities (Fig. 3A, B), we also conducted region-of-interest analyses (see below). In humans, occipitotemporal activation also comprised the striate and extrastriate cortices (e.g., MT: middle temporal area / V5, lateral occipital cortex), as well as the inferior temporal cortex (i.e., fusiform gyrus) and posterior superior temporal sulcus (pSTS), and we found activation in human somatosensory cortices (i.e., postcentral gyrus). Exclusively in humans, we observed activation in parietal cortices (i.e., superior and inferior lobules), subcortical areas such as the amygdala and thalamus, and (pre-) motor cortices (i.e., inferior frontal and precentral gyrus). In dogs, activation in the higher-order occipitotemporal association cortex remained after controlling for low-level visual stimulation (i.e., scrambled motion) or object motion (seeFig. 5Cfor the intersection of both contrasts in dogs), while humans showed activation in temporal, parietal, and somatosensory areas. Human premotor activation was only found when contrasting with the scrambled motion control (Supplementary Fig. S1), but not the object motion control (Fig. 5B).

Secondary analyses revealed that results for the dog data also remained significant using the same cluster-defining threshold of*p*< .001 as for the human data (see OSF project site for details and statistical mapshttps://osf.io/z479k/).

#### 3.1.1 Functionally analogous temporal areas sensitive to face and body perception and agents in action

Temporal cortex activation in the human action observation network has been categorized as pertaining to two distinct components: face- and body-sensitive areas in the ventral visual pathway and areas sensitive to dynamic visual aspects of social cues and action features in the lateral temporal or third visual pathway (see e.g.,Pitcher & Ungerleider, 2021;Ungerleider & Haxby, 1994;Wurm & Caramazza, 2022for reviews). To identify the functional analogues of the human face- and body-sensitive areas and disentangle them from potential functional analogues of human lateral temporal brain areas sensitive to action features in dogs, we used data from a functional localizer. This identified an area in the mid suprasylvian gyrus of dogs that was sensitive to faces and bodies and an additional area associated with body perception in the dog caudal suprasylvian gyrus (seeFig. 5D;Supplementary Note 1). These areas overlapped with the mid and caudal suprasylvian clusters in the dog AON (seeFig. 5C). However, action observation also led to activation in further temporal areas, such as the ventral caudal composite gyrus and a cluster including the rostral sylvian gyrus and ectosylvian sulcus, henceforth referred to as the rostral sylvian area.

#### 3.1.2 No differences in activation for transitive versus intransitive actions in dogs, and greater activation for dog compared with human actions in both species

Next, we tested our hypothesis of comparable engagement of the dog and human action observation network during transitive and intransitive action observation and explored how they perceived actions performed by the other species versus by conspecifics. Results revealed significant main effects of action in humans, and of agent in both species, but no significant action x agent interaction in either species (seeFig. 6;Supplementary Table S7).

**Fig. 6.**
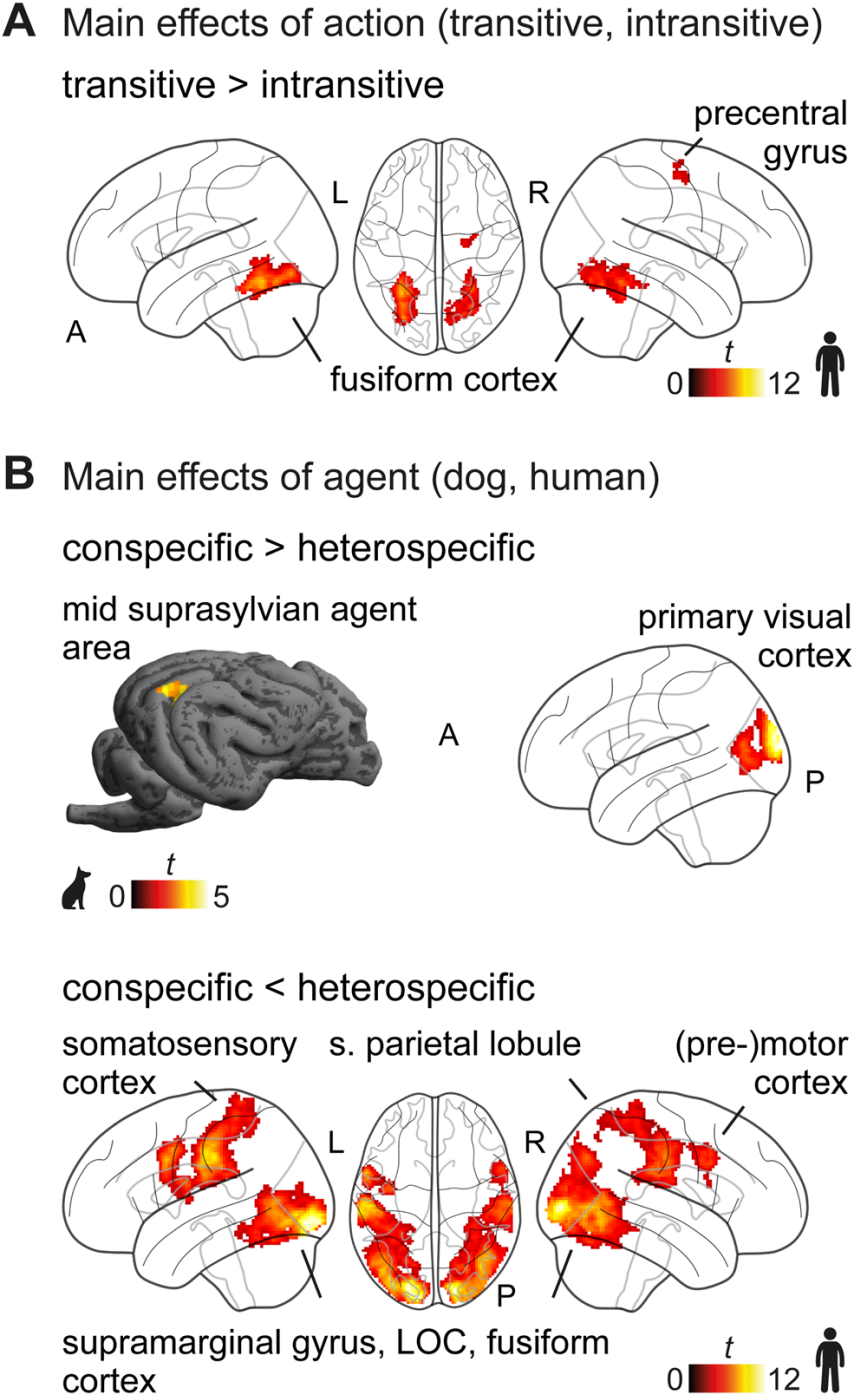
No differences in activation for transitive versus intransitive actions in dogs and greater activation for observing dog compared with human actions in both species. (A) Transitive compared with intransitive actions led to greater activation in the human inferior temporal cortex. (B) Observing conspecific compared with heterospecific actions led to greater activation in the dog mid suprasylvian agent area and human early visual cortex. The reversed contrast revealed greater activation in frontoparietal and occipitotemporal cortices in humans but no significant cluster in dogs. Results are*p*< .05 FWE corrected at cluster level using a cluster-defining threshold of*p*< .005/.001 for dogs/humans; seeSupplementary Table S7). LOC, lateral occipital cortex; A, anterior; P, posterior; L, left; R, right; s., superior,*t*,*t*-value. The icons were purchased fromhttps://thenounproject.com(royalty-free license).

In more detail, transitive compared with intransitive action observation led to greater activation in the primary somatosensory cortex (i.e., precentral gyrus) and the inferior temporal cortex in humans (Fig. 6A). We did not find significant activation for the reversed contrast. In dogs, we did not find a significant difference in activation between transitive and intransitive actions. Activation maps for each condition (compared with the implicit visual baseline) confirmed similar activation for transitive and intransitive actions in dogs (seeSupplementary Fig. S2 and S3for activation of each condition of interest > implicit baseline for both species).

Observing conspecific compared with heterospecific actions resulted in greater activation in the left mid suprasylvian animate area of dogs and the primary visual cortex of humans (Fig. 6B). The reversed contrast did not reveal any significant clusters in dogs. In humans, observing heterospecific (i.e., dog) compared with conspecific actions resulted in greater activation in clusters expanding across the frontoparietal (i.e., precentral and postcentral gyrus, bilateral supramarginal gyrus) and occipital–temporal cortices (e.g., MT, fusiform gyrus, inferior occipital gyrus), largely overlapping with the activation observed for action observation (see e.g.,Fig. 5A). Secondary analyses revealed that results for the dog data also remained significant using the same cluster-defining threshold of*p*< .001 as for the human data (see OSF project site for details and statistical mapshttps://osf.io/z479k/). Furthermore, using multiple regression analyses, we found no significant relationship between stimulus exposure (i.e., number of sessions) and activation for transitive or intransitive action observation > implicit baseline in the second task run. We also found no evidence for an effect of age, grey matter volume (mean = 65.65 mm^3^,*SD*= 4.46 mm^3^), or brain shape (i.e., neurocephalic index; mean = 65.91,*SD*= 3.01) on the activation during transitive and intransitive action observation.

### 3.2 Region-of-interest analysis reveals somatosensory but no (pre-) motor activation during action observation in dogs

The region-of-interest (ROI) analysis in the dog sensory–motor cortices yielded no significant changes in activation levels for action observation compared with object or scrambled motion in the dog pre-motor and motor cortex (seeFig. 7A;Supplementary Table S4). Visual exploration of the data revealed that only 8 out of the*N =*28 dogs displayed higher activation levels for actions than both visual controls. As expected, the temporal signal-to-noise ratio in dog frontal lobes was decreased due to air-filled cavities (i.e., sinuses) partly affecting the precruciate gyrus (i.e., premotor and secondary motor cortex; seeFig. 3C). Exploratory analyses with the seven (i.e., top 25%) dogs exhibiting the highest tSNR values in the precruciate gyrus also revealed no significant activation levels across conditions in this brain region (seeSupplementary Table S4). The ROI analysis, including data from all dogs, revealed, however, somatosensory involvement during action observation. We found greater activation for action observation compared with object motion in the dog primary somatosensory cortex (rostral suprasylvian gyrus) and greater activation for action observation compared with both controls in the dog secondary somatosensory cortex (rostral ectosylvian gyrus). Activation levels in dog sensory–motor regions were not modulated by agent or action type (seeSupplementary Table S5).

**Fig. 7.**
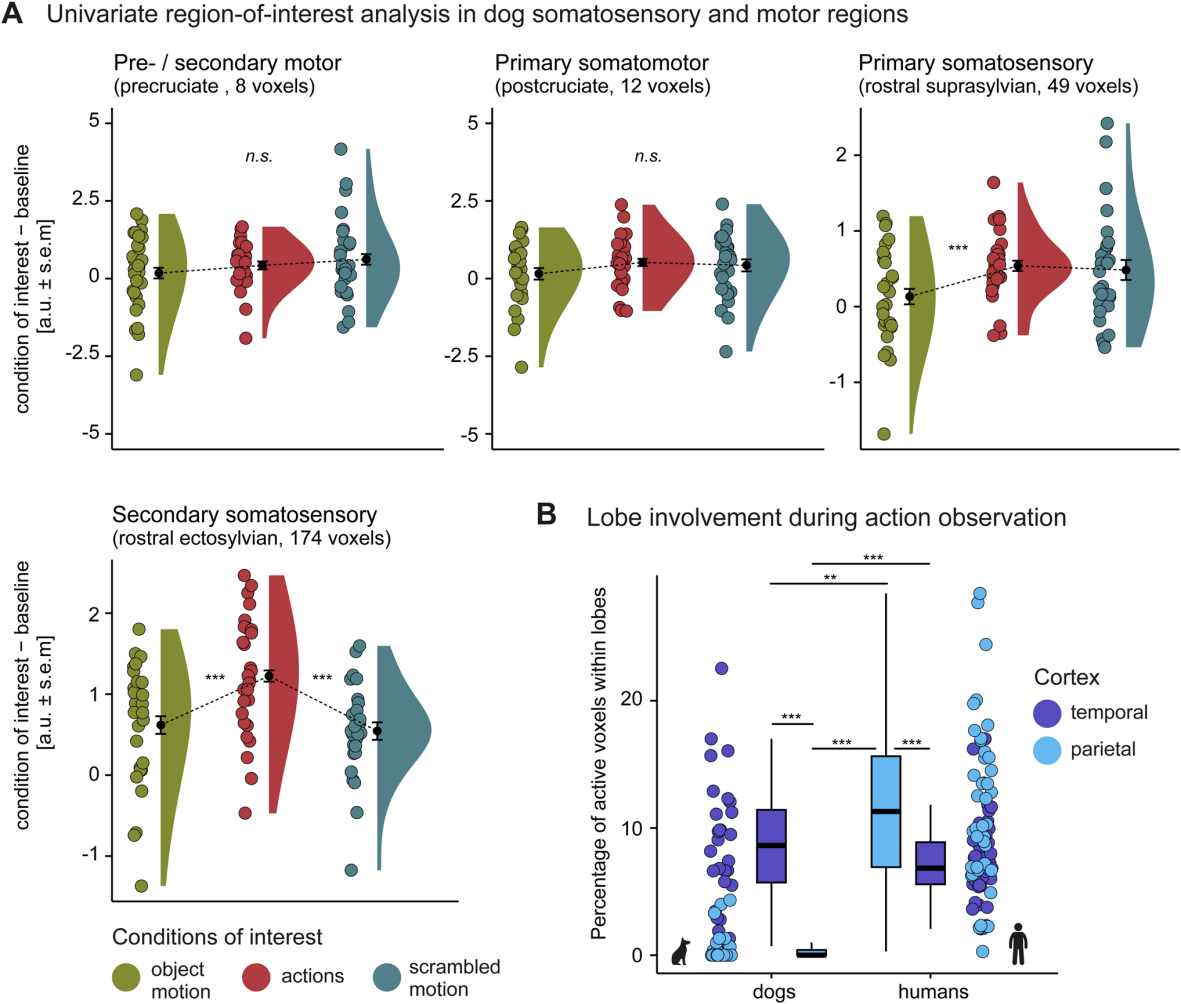
Somatosensory but no (pre-) motor activation in dogs and significantly more temporal than parietal activation in the dog compared with human action observation network. (A) Action observation compared with object and scrambled motion control did not lead to differences in activation levels in dog (pre-) motor cortices but in the rostral suprasylvian primary somatosensory and rostral ectosylvian secondary somatosensory cortex. The raincloud plots (Allen et al., 2019) show the group mean activation measured in arbitrary units (a.u.) with error bars indicating the standard error of the mean (s.e.m.), individual means (coloured dots) and density plots (half violins). (B) Action observation resulted in a significantly higher percentage of active voxels in the human compared with the dog parietal cortex. Dogs had significantly more active voxels in the temporal than the parietal cortex, while humans showed a reversed pattern. Discrepancies between lobes were more significant in dogs, with most having 0% active parietal voxels. We determined active voxels for the individual action observation contrast maps (i.e., all conditions displaying an agent acting > implicit baseline) by applying cluster-level inference (*p*< .005/.001 dogs/humans) and a cluster probability of*p*< .05 FWE corrected (see alsoSupplementary Table S6). The boxplots show the median percentage (black horizontal line), interquartile range (box), the lower/upper adjacent values (whiskers), and are accompanied by coloured dots representing the individual percentages. Planned comparisons were false discovery rate (FDR) corrected to control for multiple comparisons. **p< .01, ***p< .001; n.s., not significant. The dog and human icons in A were purchased fromhttps://thenounproject.com(royalty-free license).

### 3.3 Divergent patterns of parietal and temporal cortex engagement in dogs and humans

As the final analysis step, we tested our hypothesis of differential temporal and parietal cortex engagement in the dog and human action observation networks by first comparing the extent of activation in the two lobes within and between the two species and second the strength of task-based functional connectivity between the primary visual cortex (V1) and the temporal versus parietal cortex in dogs and humans.

#### 3.3.1 Significantly more temporal than parietal activation in the dog compared with human action observation network

We quantitatively tested the hypothesis of stronger temporal than parietal cortex activation in dogs by directly comparing the activation extent in the parietal lobes of dogs and humans. To this end, we measured the individual percentages of active voxels within each species’ lobe during action observation compared with the implicit visual baseline.

In line with our hypothesis and confirming the descriptive whole-brain findings (seeFig. 5A), the cross-species comparison revealed significantly higher proportions of active voxels in the temporal compared with the parietal lobe of dogs and significantly higher percentages of active voxels in the parietal visual and multisensory cortex of humans as compared with dogs (seeFig. 7B;Supplementary Table S6). The majority of dogs had 0% active voxels in the parietal cortex. We found the reversed pattern with greater parietal than temporal involvement in humans. However, discrepancies between the engagement of the two lobes were less pronounced, showing more distributed involvement of both lobes. Secondary analyses further confirmed that the observed between- and within-species differences in activation extent also remained using more liberal thresholds to define active voxels (seeSupplementary Fig. S4).

### 3.4 Greater task-based functional connectivity between the primary visual and temporal compared with parietal cortex in dogs

We then compared task-based functional connectivity between V1 and the temporal and parietal cortex during action observation in both species to test whether the more pronounced temporal cortex engagement in dogs was not only translated into greater activation extent but also greater functional connectivity with V1 compared with the parietal cortex within the dog action observation network.

The analysis revealed significantly greater connectivity between V1 and the temporal compared with the parietal cortex in dogs. We did not find a significant difference in connectivity in humans between the two lobes (seeFig. 8A;Supplementary Tables S8 and S9). In dogs and humans, connectivity between V1 and the temporal cortex was the strongest during action observation compared with both controls. Parietal lobe connectivity patterns diverged in the two species. V1 connectivity was significantly higher in dogs for object than for scrambled motion and action observation. In contrast, we did not find differences in connectivity for object motion and action observation in humans, but both significantly differed from scrambled motion.

**Fig. 8.**
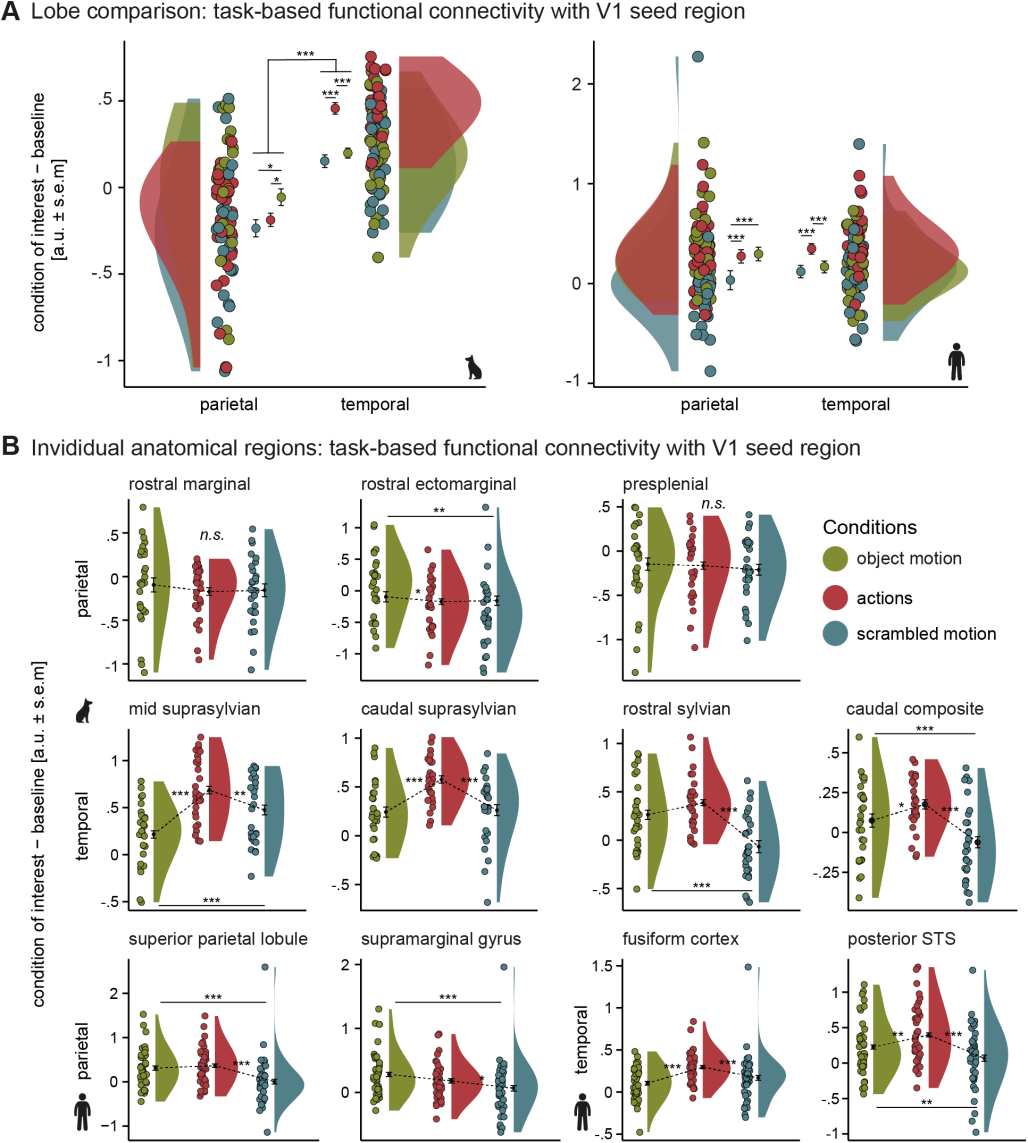
Greater task-based functional connectivity between the primary visual and temporal compared to parietal cortex in dogs. (A) Overall, we found greater task-based functional connectivity between the temporal and the primary visual cortex (V1) seed region than the parietal cortex but no differences in V1 connectivity between the two lobes in humans. In both species, connectivity between V1 and the temporal cortex was significantly greater during action observation compared to controls. In the dog parietal cortex, object motion led to higher V1 connectivity compared to scrambled motion and action observation. In humans, connectivity between V1 and the parietal cortex did not differ between action observation and object motion (Supplementary Tables S8-S9). (B) In humans, we found the same connectivity patterns observed on the lobe levels in each individual anatomical mask. This was also the case in most anatomical masks in dogs, but we did not find a significant effect of condition in the parietal rostral marginal and presplenial gyrus (Supplementary Table S10). V1 task-based functional connectivity during object motion compared to action observation did not significantly differ for the anatomical rostral sylvian mask but for the more constrained functionally defined rostral sylvian area mask (Supplementary Figure S5). The raincloud plots (Allen et al., 2019) show the group mean task-based functional connectivity with V1 measured in arbitrary units (a.u.) with error bars indicating the standard error of the mean (s.e.m.), individual means (coloured dots) and density plots (half violins). Planned comparisons were FDR corrected. *p< .05 **p< .01, **p< .001; n.s., not significant.

Next, investigating task-based functional connectivity levels of each anatomical ROI separately, we found the same connectivity patterns as observed on the lobe level in the dog parietal anterior ectomarginal gyrus and in both human anatomical ROIs (i.e., superior parietal lobule, supramarginal gyrus). We did not find a significant effect of condition in dogs’ parietal rostral marginal and presplenial gyri (seeFig. 8B;Supplementary Table S10). V1 connectivity was the highest during action observation compared with both controls in each dog and human temporal ROI, and we found significantly greater connectivity for object compared with scrambled motion in the dog rostral sylvian and caudal composite gyri, and the human posterior STS. In the dog mid suprasylvian gyrus, task-based connectivity was greater for scrambled than for object motion. The difference in connectivity between action observation and object motion did not reach significance for the anatomical rostral sylvian gyrus mask. However, using a more constrained functional mask of the rostral sylvian area derived from the univariate analysis (seeFig. 5B), we did find a significant difference in connectivity between action observation and both control conditions. All other connectivity results found with the anatomical ROIs in dogs were also replicated using the more constrained functionally defined ROIs (seeSupplementary Fig. S5andTable S10for secondary analysis). Finally, results from the localizer task showed that the agent areas also engage in greater information exchange with V1 during face and body compared with the controls, further emphasizing their functionally analogous function of human ventral temporal brain areas (seeSupplementary Note 1for details).

## 4 Discussion

The major aim of this comparative neuroimaging study was to localize the dog action observation network for the first time and to identify functionally (a) analogous and (b) divergent neural underpinnings with the human action observation network (AON). Our findings revealed multiple functional analogies between the two species, such as the involvement of temporal and somatosensory regions during action observation compared with the visual control conditions. As expected, in light of the two species’ imitation (Hoehl et al., 2019;Huber et al., 2018;Range et al., 2011) and spontaneous action matching (Fugazza et al., 2023) skills, the dog and human AONs responded to the observation of both transitive and intransitive actions with similar activation for both action types and greater activation for transitive actions only in the human inferior temporal cortex. Together with the data from a functional localizer task for the dogs, our results also confirmed our prediction of functionally analogous temporal AON components associated with (1) face and body perception and (2) processing of action features. However, we also identified expected and unexpected divergencies in the species’ AONs. As predicted, based on the differential evolutionary history and relative lobe expansion of the dog and human temporal and parietal cortex (Garin et al., 2022), we found more pronounced temporal than parietal cortex involvement in dogs during action observation in terms of the extent of active voxels and task-based functional connectivity with V1. In line with our hypothesis, based on differences in tool using behaviours and different patterns of lobe expansion, we found significantly more parietal cortex activation in humans than in dogs during action observation and more distributed involvement of temporal and parietal cortical areas within the human AON. Unexpectedly, but likely due to severe signal loss in this area in dogs, we only found premotor activation in response to action observation in humans. Thus, overall, the observed similarities and differences provide novel insights into the evolution of the neural bases of action perception and the link to relative brain expansion and social learning.

Starting with a discussion of the functional analogies, our results show that the AONs of both species include occipital–temporal regions. Observing dogs and humans performing transitive or intransitive actions elicited greater activation and functional connectivity with V1 in the temporal body and agent-sensitive areas of both species. This included the mid and caudal suprasylvian gyrus in dogs—identified using a functional agent localizer (and seeBoch et al., 2023b;Bunford et al., 2020)—and inferior temporal cortex regions in humans (see e.g.,Kanwisher, 2010). Thus, the mid and caudal suprasylvian nodes of the dog AON appear to be functional analogues of the human ventral visual stream (Ungerleider & Haxby, 1994). As expected, action observation also engaged other temporal cortex areas sensitive to the dynamic aspects of the visual social cues. In humans, this included the posterior superior temporal sulcus (pSTS), a multi-sensory association cortex involved in action and social perception (Yang et al., 2015). In dogs, the present study was the first systematic exploration of these neural bases, and we localized two regions in the temporal cortex: the caudal composite gyrus and an area in the rostral sylvian gyrus, including the rostral ectosylvian sulcus. As the human pSTS, both areas house multisensory cortex and constitute, together with auditory regions (i.e., ectosylvian gyrus), part of a pathway expanding to sensory–motor and prefrontal cortex (Kosmal, 2000;Kosmal et al., 2004). The sylvian, ectosylvian and suprasylvian gyrus, and premotor and prefrontal regions (i.e., precruciate, prorean gyrus) have also been identified as a network systematically covarying in size (i.e., grey matter volume) across breeds that correlated with numerous behavioural specializations requiring action perception of other individuals, such as herding, sport fighting, or bird flushing and retrieving (Hecht et al., 2019). The rostral sylvian gyrus and ectosylvian sulcus are also associated with social versus non-social interaction (Karl et al., 2021) and emotion perception (Hernández-Pérez et al., 2018) and these areas showed relative cortical expansion in foxes selected for tameness compared with aggression (Hecht, Kukekova, et al., 2021). This is suggestive of the rostral sylvian area of the dog AON playing a crucial role in multi-sensory social information integration, functionally analogous to the human lateral temporal visual pathway (Pitcher & Ungerleider, 2021;Wurm & Caramazza, 2022).

In line with our prediction based on the species’ imitation skills, we further observed that the dog and human AONs were similarly engaged in observing transitive and intransitive actions. We did not find a significant difference between the observation of transitive versus intransitive action observation in dogs and no pronounced differences in humans, with greater activation for transitive actions only in parts of the fusiform cortex. Since large parts of the species’ AONs evolved independently in primates and carnivorans (Kaas, 2011;Lyras, 2009), our functionally analogous findings are likely the product of convergent evolution. Research suggests that dogs have inherited the ability to copy the behaviours of others already from their wild ancestors, as their closest non-domesticated relative, the grey wolf (*Canis lupus*), possesses complex social abilities (Range & Virányi, 2014; and see e.g.,Huber et al., 2014;Range & Marshall-Pescini, 2022for review). Wolves live in family units with strong social bonds and hunt, rear their offspring, and protect their pack together (Marshall-Pescini et al., 2017;Mech & Boitani, 2003). Close cooperation, which often requires accurately and dynamically predicting others’ actions, is thus critical for survival (Range & Virányi, 2015). Therefore, the dog AON might have already evolved in their close, non-domesticated ancestors, but, as a result of domestication, is now predominantly relevant for cooperating with their human caregivers.

Contrary to marmosets (Zanini et al., 2023), a species that also displays a tendency to imitate actions of others (Voelkl & Huber, 2000,^2007^), we did not observe stronger premotor cortex activation for transitive than intransitive actions in humans. This discrepancy may be related to differences in the design of the intransitive action condition. In the present study, we removed the toy ball (i.e., the goal of the action) from the transitive action to create the intransitive action condition, such that both conditions showed identical reach-to-grasp movements. By contrast, most primate mirror neuron localizers employ mimed reach-to-grasp actions or reach actions that stop before grasping occurs (see e.g.,Nelissen et al., 2011;Sliwa & Freiwald, 2017), the latter being used byZanini et al. (2023)in marmosets. Thus, although very similar, the kinematics and the performed actions differed between transitive and intransitive actions in previous studies, while our design kept them identical. Considering that premotor activation has been linked to processing action kinematics (Candidi et al., 2008;Pobric & Hamilton, 2006), the absence of differences in premotor activation in our study may be because the kinematics of transitive and intransitive actions were kept identical in our study. The fact that the two conditions only differed in the presence of the toy ball might also explain why we found greater activation in the fusiform cortex—an area associated with agent and object perception (Ayzenberg & Behrmann, 2022;Ungerleider & Haxby, 1994)—for transitive compared with intransitive actions, but not in temporal cortex areas associated with the processing of action features such as the posterior STS (Pitcher & Ungerleider, 2021;Wurm & Caramazza, 2022). Overall, these findings support the hypothesis that action observation in species with a tendency to imitate actions focuses more on the action itself than the action outcome or goal.

Our findings also revealed important divergencies in the neural bases of action observation, which are of particularly conceptual relevance. Based on prior research with non-human primates (Hecht, Murphy, et al., 2013;Sliwa & Freiwald, 2017;Zanini et al., 2023), we predicted a notable discrepancy in parietal cortex involvement between dogs and humans. Indeed, parietal cortex activation was significantly lower in dogs than in humans during action observation, with most dogs not having any active voxels in parietal regions. Interestingly, although to a lesser degree in humans, we also found contrasting patterns of relative lobe involvement in the two species. Dogs displayed pronounced temporal cortex engagement with significantly more active voxels in the temporal than in the parietal cortex, and task-based connectivity between V1 and the temporal cortex exceeded the connectivity with the parietal cortex. Humans had more active voxels in the parietal than in the temporal cortex, but we did not find a difference in task-based functional connectivity between the two lobes. The species’ differences align with their differential pattern of relative lobe expansion (Garin et al., 2022). As common marmosets, who also exhibit more pronounced temporal than parietal cortex activation during action observation (Zanini et al., 2023), dogs have more expanded occipital and temporal than parietal and frontal lobes. While humans, apes, and Old World Monkeys display an opposing trend with significantly more expanded parietal and frontal lobes, rhesus macaques and chimpanzees have stronger frontoparietal than temporal cortex activation during action observation (Hecht, Murphy, et al., 2013;Sliwa & Freiwald, 2017). Thus, parietal areas may play a more prominent role for action observation in species with more expanded parietal cortex.

The inferior parietal lobule (IPL)—a key region of the human, chimpanzee, and macaque AON (Fogassi et al., 2005;Hecht, Gutman, et al., 2013;Hecht, Murphy, et al., 2013;Urgen et al., 2019)—is associated with the planning and execution of reach-to-grasp actions, as well as guiding visual attention (“vision-to-action”; seeCulham & Valyear, 2006;Husain & Nachev, 2007for reviews), and the anterior supramarginal gyrus portion of the human IPL has been linked to their complex tool-using abilities (Peeters et al., 2009; and seeOrban & Caruana, 2014;Stout & Hecht, 2017for reviews). A potential explanation for the differential areal activation patterns could, therefore, be the species’ tendency for manual object interactions (i.e., with their upper limb). In marmosets, action observation did not elicit strong IPL activation with no difference in activation between transitive and intransitive action observation (Zanini et al., 2023), and unlike rhesus macaques, chimpanzees, or humans, they primarily explore and grasp objects with their mouth rather than their upper limbs (Stevenson & Poole, 1976). Dogs do not display any tool-using behaviour, and they can only grasp objects with their snout, which they mainly use to explore their environment. This suggests that the less pronounced IPL and parietal cortex involvement in marmosets (Zanini et al., 2023) and the absence of parietal cortex activation in dogs during action observation might be linked to the less evolved or absent manual object-manipulating behaviours of the species.

Moreover, the differences in manual dexterity may have contributed to differential expansion of the parietal cortex (see, e.g.,Garin et al., 2022), which in turn could account for the observed differences in activation. Research mainly within the primate lineage indeed suggests a coevolution between complex manual object-manipulating behaviours and (posterior) parietal lobe expansion and complexity (Cheng et al., 2021;Goldring & Krubitzer, 2017;Maravita & Romano, 2018). Furthermore, a systematic comparison across mammalian species found greater relative expansion of the parietal cortex in rhesus macaques, chimpanzees, and humans compared with dogs, marmosets, and other mammals that do not exhibit these behaviours (Garin et al., 2022). Investigations of regional variations in brain size are still scarce within the carnivoran lineage. First observations indicate cortical expansion and more complex sulcal configurations within the parietal cortex in carnivoran species exhibiting manual object manipulation behaviours—such as raccoons, red pandas, and other species with high forepaw dexterity—compared with species with low forepaw dexterity, such as canids or felids (Boch, Karadachka, et al., 2024;Manger et al., 2002). However, further research is needed to explore relative lobe expansion and contributing factors in carnivorans. Overall, our findings provide new insights into the relationship between parietal cortex involvement during action observation and the occurrence of complex manual object-manipulating behaviours.

Regarding sensory–motor involvement during action observation, we found partly analogous but also unexpected divergent results. As hypothesized, observing actions performed by conspecifics or heterospecifics elicited activation in human primary and secondary sensory–motor cortices (e.g., inferior frontal gyrus, post-/ precentral gyri (Caspers et al., 2010;Fabbri-Destro & Rizzolatti, 2008). In dogs, results also revealed activation in the primary (S1) and secondary (S2) somatosensory cortex (i.e., rostral suprasylvian and ectosylvian gyrus (Adrian, 1941;Pinto Hamuy et al., 1956). However, despite showing a trend in the direction, S1 activation did not significantly differ from activation for scrambled motion. Contrary to the results in humans, we did not find significant activation in the dog premotor cortex during action observation compared with the control conditions. We only found higher activation for actions compared with both controls in 8 out of the 28 dogs, including mixed-breed and 3 different pure-breed dogs. It is, therefore, unlikely that specific breeding purposes or behavioural specializations drove the observed differences in activation. It should be noted, though, that the findings on (pre)motor areas need to be interpreted with caution, as the signal in all dogs’ frontal lobes was affected by their large air-filled sinuses bordering this brain area, resulting in low tSNR values and partial up to complete signal drop out in many dogs (seeBoch et al., 2024, for a detailed discussion of this prevailing limitation in dog fMRI and potential solutions to overcome it in future work). Furthermore, although overall low, dogs had higher scan-to-scan motion than humans, which could have additionally negatively affected tSNR (Van Dijk et al., 2012). Considering this issue and the fact that mirroring activation has been demonstrated in other mammalian and bird species via cell recordings (Carrillo et al., 2019;Fujimoto et al., 2011;Hamaguchi et al., 2014;Prather et al., 2008;Viaro et al., 2021), we cannot conclude that dogs do not have premotor areas with mirroring properties. Advances in dog electroencephalography (EEG) research (Kis et al., 2014;Kujala et al., 2020;Törnqvist et al., 2013) might overcome the limitations of MRI and provide more insights into the involvement of dog sensory–motor and frontal cortices during action observation.

It is important to interpret observed activation not only as isolated localized activations but also how these “blobs” might form a network, as well as if the absence of activation in one area might be linked to a lack of activation in another. Although to varying degrees, action observation elicits activation in the parietal cortex of humans and other primates (see e.g.,Fabbri-Destro & Rizzolatti, 2008;Hecht, Murphy, et al., 2013), including common marmosets (Zanini et al., 2023). Given that the parietal cortex and premotor regions of primates are densely interconnected and play a crucial role in sensory and visuomotor integration during action observation and execution (Glover et al., 2012;Hecht et al., 2015;Hecht, Gutman, et al., 2013), one might hypothesize that the absence of parietal and premotor activation in dogs may be linked. Initial research suggests that the frontoparietal networks of dogs are more developed than those of domestic cats (Jacqmot et al., 2017) but less dominant than the ones observed in primates and humans (Szabó et al., 2019). Considering the less evolved frontoparietal connections and lack of visual parietal cortex activation in dogs during action observation, it might, therefore, not seem surprising that we did not observe activation in premotor regions. However, unlike in primates, where there are strong anatomical connections linking the premotor and parietal regions (Hecht, Gutman, et al., 2013), the frontal connections from visual regions in the parietal cortex of the dog brain do not reach the premotor region (i.e., areas in the precruciate gyrus;Markow-Rajkowska & Kosmal, 1987). This could suggest that the premotor–parietal system that is involved in action observation in primates might not exist analogously in dogs, which would, in turn, explain why we did not observe premotor activation. There are, however, strong projections between the temporal and (pre-) motor and frontal regions in the dog brain (Kosmal, 2000;Markow-Rajkowska & Kosmal, 1987), with the densest projections from the premotor cortex to mid and anterior sylvian gyrus and ectosylvian sulcus areas and, to a lesser extent, to the mid and caudal suprasylvian sulcus. The same temporal cortex regions were also active during action observation in dogs. Hence, considering the connections between the premotor and temporal regions in the dog brain, one should expect premotor activations during action observation. While further studies are necessary to understand canine brain organization better, the evidence from tracer studies, combined with our observations, emphasizes a more prominent role of the temporal cortex, in concert with frontal regions, during action observation and suggest that dog frontal–temporal pathways may take over functions associated with frontoparietal pathways in primates.

However, the question remains whether dogs perceive intentions behind actions and which brain area might be involved in encoding them. Although the AONs of common marmosets and dogs share many similarities, it is important to note that action observation compared with the implicit visual baseline resulted in IPL activation in marmosets (Zanini et al., 2023), and this area is typically also associated with encoding the intentions of actions (Fogassi et al., 2005;Patri et al., 2020) in the primate and human AONs. Results of recent behavioural research investigating dogs’ ability to attribute intentions to human or conspecific actions appear to differ depending on whether the actions are within a social or non-social context. Research on domestic dogs shows that they are sensitive to humans’ intentions in social settings, such as distinguishing between a clumsy human and one who is unwilling to cooperate (Völter, Lonardo, et al., 2023). This suggests that dogs can discern the intentions behind similar actions, but findings remain inconclusive regarding dogs’ focus of attention during the perception of object-directed actions. Eye-tracking research with dogs revealed anticipatory looking behaviours towards the target when humans performed a grasping action (Lonardo et al., 2023). However, when conspecifics performed a similar action, the dogs’ attention shifted towards the conspecific, suggesting that the agent, rather than the inanimate target, was more salient in capturing their attention. More recent evidence (Lonardo et al., 2024) also challenges previous findings (Marshall-Pescini et al., 2014), which indicated that dogs focus on the action’s goal (i.e., inanimate object). Instead, these new findings suggest that dogs focus more on the movement trajectory of the action itself rather than the inanimate action target. Considering that motion trajectories of the transitive and intransitive actions did not differ in our study, the behavioural evidence aligns with our results, showing no significant differences in activation between the two conditions. However, the rostral sylvian gyrus, one of the identified action areas in the dog temporal cortex, might be sensitive to encoding intentions in social interactions, as it has been shown to respond differently to positive (i.e., caressing) compared with neutral (i.e., ear or mouth assessment) human–dog interactions (Karl et al., 2021). More studies are now needed to investigate the roles of the identified nodes within the dog AON further.

Unlike the comparisons to scrambled motion or the implicit visual baseline, action observation compared with object motion did not elicit activation in the premotor cortex or the inferior parietal lobule in humans. Furthermore, comparing activation for each condition with the implicit baseline shows that action observation and object motion recruit frontoparietal nodes of the human AON. Similarly, task-based connectivity between V1 and the parietal cortex did not differ between object motion and action observation, but was higher for both conditions than for scrambled motion. The reason for this observation may lie in the intrinsic characteristics of the motion control itself. In our task, the object motion control was created by removing the agent from the grasping video, leaving the object (i.e., toy ball) to follow the original biologically plausible trajectory driven by the now invisible agent. Previous research in humans demonstrated that clouds of dots moving with kinematics similar to hand movements engage frontoparietal regions overlapping with the human AON (Dayan et al., 2007). Furthermore, seeing the agents grasping objects in the transitive condition videos likely allowed human participants to infer the action performed with the object (i.e., one of its affordances) when they only saw the object, and participants might have even attempted to predict or imagine the invisible action. It has been shown that the mere presentation of an object, such as a toy ball, can already elicit activation in frontoparietal regions as object affordances are processed (see e.g.,Maranesi et al., 2014for review). Thus, the strong frontoparietal engagement during object motion perception in humans might be the result of processing both biological motion and object affordances.

Lastly, due to their close social bond (Archer, 1997;Topál et al., 1998), we also explored how dogs and humans perceive each other compared with conspecifics. Interestingly, observing conspecific compared with heterospecific actions elicited increased activation only in human primary visual cortices (V1). However, the reversed contrast led to activation in the entire human AON. Less familiarity with actions performed by heterospecific compared with conspecifics and, for humans, uncommon use of the mouth to pick up an object might require increased engagement of the action observation network to understand or predict the observed action, which would align with the predictive coding account of action observation (Kilner et al., 2007). The stronger activation could also be the result of heightened attention or interest towards dogs compared with humans. As the value-driven model (Aziz-Zadeh et al., 2018) adds to the predictive coding account, engagement of the action observation network is modulated by personal interest or value. To the best of our knowledge, only one prior human fMRI study investigated the neural bases while observing actions performed by dogs or humans. However, that study did not directly contrast dog versus human action (Buccino et al., 2004). Hence, more neuroimaging research is needed to explore the neural bases of heterospecific action perception in humans further. In dogs, we found no significant activation increases in response to human compared with conspecific actions. The reversed contrast only led to significantly more activation in the mid suprasylvian animate area, which has been previously associated with higher sensitivity towards conspecifics (Bunford et al., 2020). The lack of pronounced differences between the perception of dog and human actions may be surprising at first sight. Note, however, that all pet dogs participating in our study grew up in human households and are, therefore, highly familiar with human actions, and already dog puppies spontaneously imitate human actions (Fugazza et al., 2023). Thus, dogs’ attention towards human social cues has likely been enhanced through domestication (Bray et al., 2021), and their AON is, therefore, potentially tuned to human and dog actions.

As this was the first study investigating the dog action observation network, we utilized a well-controlled task design commonly employed for mirror neuron localizers in primate research (e.g.,Nelissen et al., 2005,^2011^;Sliwa & Freiwald, 2017). Building on our work, future comparative studies need to explore the involvement and interplay of the dog and human AON nodes using more naturalistic stimuli, such as movie-based studies (Hori et al., 2021;Mantini et al., 2012;Richardson & Saxe, 2020). Another avenue could be the use of event-related design to minimize potential effects of repetition suppression (Kilner et al., 2009), though this may come at the cost of less statistical power compared with block designs. Importantly, given that we applied the same stringent criteria to monitor the dogs’ attention in both tasks, it is unlikely that the differences in activation between the agent localizer and action observation merely reflect differences in attention towards static versus dynamic stimuli. Furthermore, a recent comparative neuroimaging study investigating agent perception in dogs and humans using dynamic stimuli (e.g., videos with minor head movements, eye blinks or changes in gaze direction but no targeted actions) also identified the same agent-sensitive regions as our localizer (i.e., mid and caudal suprasylvian gyrus;Bunford et al., 2020). They do not report activation in the action-sensitive areas revealed by our action observation study. This further emphasizes that the identified mid and caudal suprasylvian nodes of the dog AON are likely functional analogues of the human ventral visual stream (Ungerleider & Haxby, 1994), while the other AON nodes in the dog temporal cortex are involved in action perception.

In conclusion, our study marks the first investigation of the dog action observation network. The analogous engagement of somatosensory and temporal areas suggests that both species evolved partly similar networks engaged in action observation. The strong differences in parietal lobe involvement provide exciting new insights into how differential cortical expansions may support brain functions, speaking for the divergent evolution of the species’ object-manipulating behaviours. The findings and our overall approach provide strong foundations and the first stepping stone for future studies investigating the evolutionary history of primate and canine action perception. We hope this will ultimately inform a more advanced understanding of the evolution of the neural bases of social behaviour and learning.

## Supplementary Material

Supplementary Material

## Data Availability

We analysed the data using Matlab 2020b (MathWorks), SPM12 (https://www.fil.ion.ucl.ac.uk/spm/software/spm12/), and R 4.3.0 (R Core Team, 2023). We employed all linear mixed models using the R packages*lme4*(Bates et al., 2015) and*afex*(Singmann et al., 2020), and created the figures using the R packages ggplot2 (Wickham, 2016) and RainCloudPlots (Allen et al., 2019), and the python project nilearn (http://nilearn.github.io), and MRIcron (https://www.nitrc.org/projects/mricron). The task was implemented using PsychoPy (Peirce, 2007). The data supporting this manuscript are openly available on the Open Science Framework (OSF;https://osf.io/z479k). This includes beta maps of all univariate group comparisons, all functionally defined region-of-interest (ROI) masks, detailed sample descriptives, individual motion parameters, raw ROI data (univariate and gPPI), dog whole-brain temporal signal-to-noise ratio maps, and the stimulus material. Raw dog neuroimaging data are publicly available at Zenodo (https://doi.org/10.5281/zenodo.13860616). Due to ethical constraints, raw human neuroimaging data are made available from the lead author upon request. Custom R code supporting this manuscript is publicly available at the study’s OSF repository (https://osf.io/z479k).
